# Coordinated electrical activity in the olfactory bulb gates the oscillatory entrainment of entorhinal networks in neonatal mice

**DOI:** 10.1371/journal.pbio.2006994

**Published:** 2019-01-31

**Authors:** Sabine Gretenkord, Johanna K. Kostka, Henrike Hartung, Katja Watznauer, David Fleck, Angélica Minier-Toribio, Marc Spehr, Ileana L. Hanganu-Opatz

**Affiliations:** 1 Developmental Neurophysiology, Institute of Neuroanatomy, University Medical Center Hamburg-Eppendorf, Hamburg, Germany; 2 Department of Chemosensation, Institute of Biology II, Rheinisch-Westfälische Technische Hochschule Aachen, Aachen, Germany; Institute of Science and Technology Austria, Austria

## Abstract

Although the developmental principles of sensory and cognitive processing have been extensively investigated, their synergy has been largely neglected. During early life, most sensory systems are still largely immature. As a notable exception, the olfactory system is functional at birth, controlling mother–offspring interactions and neonatal survival. Here, we elucidate the structural and functional principles underlying the communication between olfactory bulb (OB) and lateral entorhinal cortex (LEC)—the gatekeeper of limbic circuitry—during neonatal development. Combining optogenetics, pharmacology, and electrophysiology in vivo with axonal tracing, we show that mitral cell–dependent discontinuous theta bursts in OB drive network oscillations and time the firing in LEC of anesthetized mice via axonal projections confined to upper cortical layers. Acute pharmacological silencing of OB activity diminishes entorhinal oscillations, whereas odor exposure boosts OB–entorhinal coupling at fast frequencies. Chronic impairment of olfactory sensory neurons disrupts OB–entorhinal activity. Thus, OB activity shapes the maturation of entorhinal circuits.

## Author summary

Cognitive performance is maximized only through permanent interactions with the environment, yet the contribution of sensory stimuli to cognitive processing has been largely neglected. This is especially true when considering the maturation of limbic circuits accounting for memory and executive abilities. Rodents are blind and deaf, do not whisker, and have limited motor abilities during the first days of life, and therefore, the contribution of sensory inputs to limbic ontogeny has been deemed negligible. As a notable exception, olfactory inputs are processed already early in life and might shape the limbic development. To test this hypothesis, we investigate the principles of communication between the olfactory bulb (OB), the first processing station of olfactory inputs, and lateral entorhinal cortex (LEC)—the gatekeeper of limbic circuits centered on hippocampus and prefrontal cortex—of mice during the first and second postnatal weeks. We show that spontaneously generated patterns of electrical activity in the OB activate the entorhinal circuits via mono- and polysynaptic axonal projections. The activity within the circuitry connecting the OB to the LEC is boosted by odors and disrupted by chronic lesion of the olfactory periphery. Thus, spontaneous and stimulus-induced activity in the OB controls the maturation of neuronal networks in the LEC.

## Introduction

Coordinated patterns of electrical activity entrain developing neuronal networks in rhythms with a broad frequency spectrum. These patterns have been proposed to critically shape brain maturation [1–3]. Experimental evidence supporting this hypothesis has been mainly provided for sensory systems. For example, in the visual and auditory systems, spontaneous activity from sensory periphery (i.e., retina or cochlea) controls the formation of cortical representations underlying stimulus perception [4,5]. Theta band (4–12 Hz) spindle bursts and gamma (30–80 Hz) oscillations in the developing somatosensory system promote thalamocortical connectivity and maturation of coupling with the motor system [6,7]. Overall, the discontinuous oscillatory activity in sensory cortices during development has multifold origin, including stimulus-independent activation in the periphery and entrainment of local cortical circuits via chemical and electrical synapses [1,8].

Although less investigated, limbic circuits show similar patterns of coordinated activity during early development, with discontinuous theta bursts (4–12 Hz) and superimposed fast frequency episodes (20–40 Hz) [9–13]. Theta bursts facilitate unidirectional communication from the CA1 area of intermediate/ventral hippocampus (HP) to the prelimbic subdivision of the prefrontal cortex (PFC) via glutamatergic projections [14]. As a consequence of hippocampal theta drive, pyramidal neurons in local prelimbic circuits generate beta–low gamma (20–40 Hz) oscillations [15]. Theta coupling between neonatal PFC and HP is controlled by the lateral entorhinal cortex (LEC), which densely projects to both areas [11]. The complex organization of limbic circuits at an early age raises the question of which mechanisms control the gatekeeper function of LEC during early development. Similar to sensory systems, the neonatal LEC might be driven by spontaneous activity from the sensory periphery. Indeed, the adult LEC receives direct input from the olfactory bulb (OB), which, in contrast to other sensory systems, bypasses the thalamus [16,17]. Mitral and tufted cells (MTCs) represent the sole OB output neurons. Rather than simply relaying information, these neurons are embedded in a complex network that controls odor information coding [18,19]. The axons of mitral cells (MCs) terminate in entorhinal layer I on apical dendrites of layer II/III pyramidal and stellate cells [20], which in turn form the perforant path projection to the hippocampal formation [21,22]. Layer II/III neurons in LEC project back to OB [23], yet distinct entorhinal populations are differently engaged in feedforward and feedback signaling during odor processing [24]. Thereby, odor-evoked activity in the adult controls the gateway function of LEC, which interfaces HP and neocortical regions [25,26].

Whereas the sense of smell serves fundamental functions in newborn animals [27], the role of olfactory inputs and OB activity for limbic circuit maturation remains unknown. Since other sensory systems are still immature during early life—and thus their impact on limbic circuits is negligible—this knowledge gap appears even more striking. Rodent pups are blind and deaf and have limited sensorimotor abilities until the end of the second postnatal week [28,29]. In contrast, the olfactory system that provides the major sensory input in neonatal rodents maturates earlier, yet it permanently evolves during the first 2 postnatal weeks [30]. We hypothesize that both odor-dependent and odor-independent coordinated activity in OB control the entrainment of entorhinal networks during neonatal development. Combining anatomical tracing with optogenetics, electrophysiology, pharmacology, and sensory manipulation in urethane-anesthetized and awake neonatal mice (postnatal day [P]8–10) in vivo, we elucidate the olfactory control of functional maturation of entorhinal circuits.

## Results

### OB and LEC are reciprocally connected in neonatal mice

**Fig 1:**
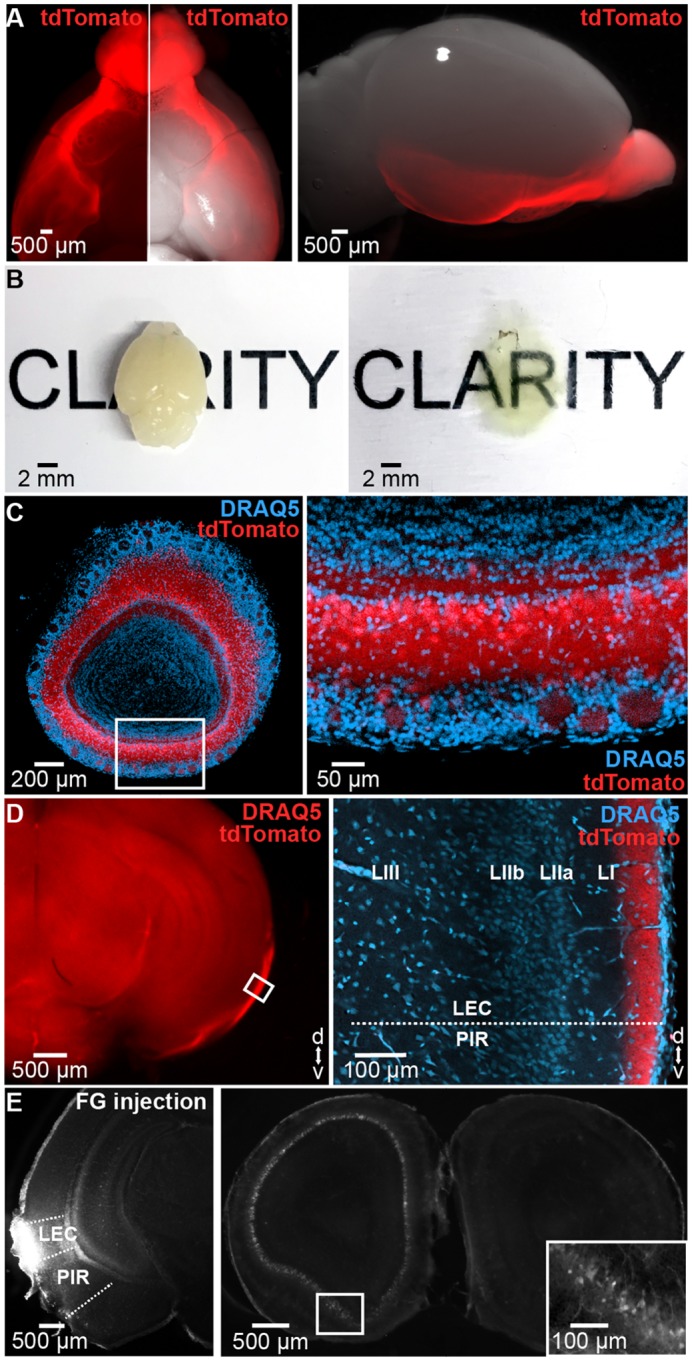
Bottom-up connectivity between OB and LEC in neonatal mice. (A) Long-range projections of tdTomato fluorescently labeled OB MTCs (left) when superimposed on a bright-field image showing the ventral (middle) and lateral (right) view of the whole brain of a P10 Tbet-cre;tdTomato mouse. (B) Unprocessed (left) and cleared (right) brain of a P10 mouse. (C) Cleared 500 μm–thick coronal section containing the OB of a Tbet-cre;tdTomato mouse showing MTCs (red) when counterstained with the nuclear marker DRAQ5 (blue). Inset, tdTomato-stained MTCs displayed at larger magnification. (D) MTC axons targeting LEC in a cleared 1 mm–thick coronal brain slice. Inset, axons of tdTomato-expressing MTCs when counterstained with DRAQ5 (blue) and displayed at larger magnification. (E) Photographs of a 100 μm–thick coronal section from a P8 mouse depicting retrogradely labeled neurons in the OB (right) after injection of FG into the LEC (left) at P3. Inset, FG-labeled MTCs displayed at larger magnification. FG, Fluorogold; LEC, lateral entorhinal cortex; MTC, mitral and tufted cell; OB, olfactory bulb; P, postnatal day; PIR, piriform cortex.

In mice, MTCs mature during intrauterine life, and their axons reach cortical targets during the first postnatal week [30]. This time window coincides with the period of strong gating of prefrontal-hippocampal networks by entorhinal theta activity. To detail the spatial patterns of connectivity between OB and LEC in P8–10 mice, we performed an in-depth investigation of axonal projections from MTCs to LEC and, vice versa, of entorhinal projections to OB. First, we used Tbet-cre;R26-tdTomato mice (*n* = 4) for intact-brain imaging of long-range projections by electrophoretic tissue clearing and confocal fluorescence microscopy (Fig 1A and 1B). In these mice, MTCs are genetically tagged (Fig 1C) [31]. Already at P8, the lateral olfactory tract (LOT) comprising MTC axons reached the posterior part of the cerebrum, including piriform cortex (PIR) and LEC (Fig 1A and 1D). As previously shown in adult rats [20], MTC axons were mainly confined to layer I of neonatal LEC (Fig 1D). Retrograde tracing with Fluorogold (FG) injected into LEC of P3–4 mice confirmed the direct connectivity (Fig 1E). No differences between dorsal and ventral OB were detected with respect to the density of MTC projections to LEC.

Second, we assessed the spatial organization of feedback projections from LEC to OB. Unilateral injection of FG confined to OB of P3–4 mice (*n* = 12) led to bright fluorescent back-labeling of parental cell bodies in ipsilateral LEC that project to OB of P8–10 mice (S1A Fig). Their density was lower when compared to the cells detected in ipsilateral PIR (S1A Fig). Most labeled neurons were located in layer II and III (88.40%, 259/293, 3 pups, 11 sections). To examine the neurochemical identity of entorhinal neurons projecting to OB, we counterstained the LEC sections containing FG-labeled neurons for CamKII and GABA. CamKII staining revealed that the large majority but not all FG-labeled cells were glutamatergic (S1B Fig). GABA staining confirmed these results. Whereas most OB-projecting neurons (99.66%, 292/293) were negative for GABA, hence glutamatergic, a very small fraction (0.34%, 1/293) was GABA-positive. These data indicate that top-down projections from LEC to OB are mainly excitatory. The GABAergic projections seem to augment their density with age [32].

Taken together, the results of morphological investigation show that, similar to connectivity in adult mice [33,34], afferent and efferent projections couple neonatal LEC and OB. Whereas glutamatergic MTC axons target entorhinal layer I, glutamatergic and very few GABAergic neurons in superficial layers of LEC innervate the developing OB.

### Continuous respiration-related activity and discontinuous theta bursts entrain the neonatal OB

**Fig 2:**
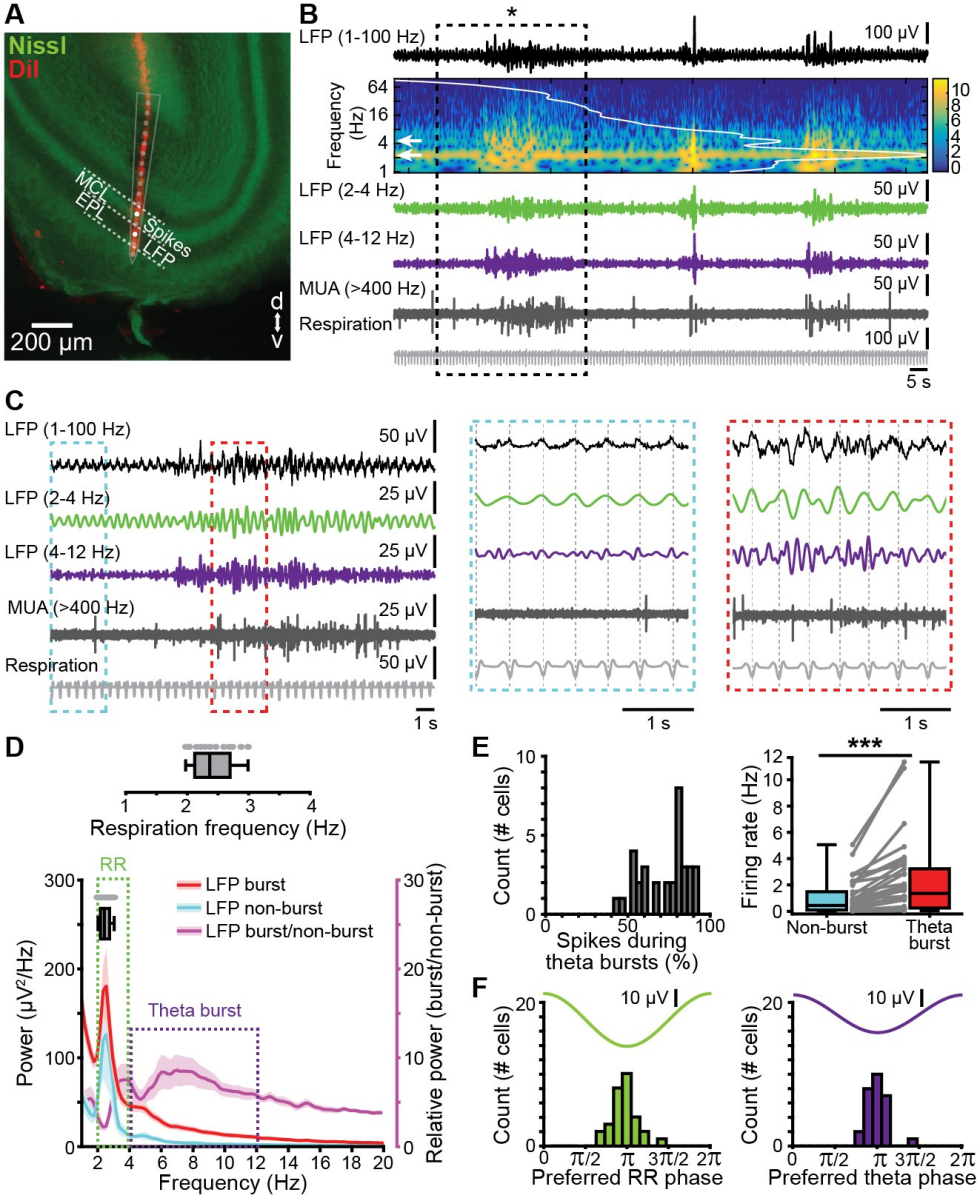
Continuous and discontinuous patterns of oscillatory activity in the neonatal OB. (A) Digital photomontage reconstructing the track of the DiI-labeled multisite recording electrode (red) in a Nissl-stained (green) 100 μm–thick coronal section including the OB from a P9 mouse. The dots (gray) show the position of the 16 recording sites of the silicon probe and the recording channels (white) in the MCL and EPL that were used for spike and LFP analysis, respectively. (B) LFP recording of the oscillatory activity in the OB of a P10 mouse displayed band-pass filtered in different frequency bands and accompanied by the wavelet spectrogram (white line represents time-averaged power of the trace; white arrows point toward peak frequency values) as well as simultaneously recorded MUA (high-pass filter >400 Hz) and respiration. (C) Characteristic slow continuous oscillatory activity and theta bursts from the trace shown in B when displayed at higher magnification. Insets, continuous oscillatory activity in relationship with respiration (left, blue) and a discontinuous theta burst (right, red). (D) Power spectra (mean ± SEM) of LFP in OB during nonburst activity (blue) and discontinuous bursts (red) as well as of theta bursts normalized to nonbursting activity (purple). The respiration frequency was depicted as a horizontal bar and expanded at larger scale (top). (E) Temporal relationship between neuronal firing and network oscillations in OB. Left, histogram showing the percentage of spikes occurring during theta burst for all clustered units. Right, box plot depicting the firing rates of OB units during nonburst periods and theta burst periods. Gray dots and lines correspond to individual cells (Wilcoxon signed-rank test, ***p < 0.001). (F) Histograms depicting the phase locking of OB cells to RR (left) and theta activity (right). Only significantly locked cells were used for analysis. Data are available in S1 Data. EPL, external plexiform layer; LFP, local field potential; MCL, mitral cell layer; MTC, mitral and tufted cell; MUA, multiunit activity; OB, olfactory bulb; P, postnatal day; RR, respiration-related rhythm.

Despite abundant data on morphological development, the functional maturation of OB is still largely unknown. In contrast to the retina and cochlea, which lack stimulus sensitivity at early stages of postnatal development and only generate spontaneous activity, the OB processes olfactory input already at birth [27]. To elucidate the patterns of activity in the neonatal OB, we performed multisite extracellular recordings of local field potential (LFP) and multiunit activity (MUA) from the MC layer (MCL) in the dorsal and ventral OB of P8–10 mice in vivo (*n* = 148). Unless stated otherwise, data were obtained under light urethane anesthesia. The signal reversal between the internal plexiform layer (IPL) and external plexiform layer (EPL) (S2B and S2C Fig) as well as the large MC spikes served as physiological markers for confirming the position of the recording electrode set according to stereotaxic coordinates. In addition, the location of DiI-labeled electrodes was confirmed after histological investigation post mortem (Fig 2A, S2A, S3A and S3B Figs).

Two patterns of coordinated activity were detected in OB (Fig 2B and 2C). First, we recorded continuous low-amplitude oscillations with slow-frequency peaking at 2–4 Hz. Given their temporal correlation and frequency overlap with respiration (median frequency: 2.37 Hz, interquartile range [iqr]: 2.12–2.70 of chest movements) (Fig 2C and 2D), we defined this activity as respiration-related rhythm (RR). The RR reversed over the MTC layer and had larger amplitudes in EPL and glomerular layer when compared to the activity in MTC layer (S2B Fig). Its temporal relationship to the phase of the respiratory cycle differed between layers: the rising phase of the RR cycle in the granule cell layer (GCL) and the falling phase in EPL and glomerular layer correlated with exhalation (S2B Fig). Second, we recorded discontinuous high-amplitude oscillatory events with spindle shape in the neonatal OB (Fig 2B and 2C). These events had frequencies peaking within theta frequency band (4–12 Hz) (Fig 2D) and showed a signal reversal between GCL and EPL (S2C Fig). Given their resemblance in shape and frequency dynamics to previously characterized oscillatory events in neonatal cortical areas [9–11,35], these events were classified as theta bursts.

As reported for adult OB, prominent spiking characterized neonatal MTCs. Analysis of single-unit activity (SUA) after principal component analysis (PCA)-based sorting of units revealed that the majority (80%) of spikes occurred during theta bursts. The firing rate during bursts (median: 1.36 Hz, iqr 0.25–3.23 Hz) was significantly (*p* = 3.65 × 10^−7^, Wilcoxon signed-rank test, *n* = 34 cells from 14 animals) augmented when compared to nonbursting periods (median: 0.44 Hz, iqr 0.09–1.48 Hz) (Fig 2E). To assess the temporal relationship between oscillatory OB rhythms and MTC firing, we estimated the coupling strength between SUA and RR as well as between SUA and theta bursts by calculating the pairwise phase consistency (PPC), a bias-free measure of rhythmic neuronal synchronization [36]. Both rhythms similarly timed MTC firing (RR: median PPC: 0.21, iqr 0.20–0.22 versus theta burst: median PPC: 0.21, iqr 0.20–0.21, *p* = 0.1664, Wilcoxon signed-rank test, 2 outliers removed, *n* = 32 cells) to the oscillatory trough (Fig 2F).

In adults, dorsal and ventral OB subdivisions have distinct physiology and function. MTC axons that originate in the dorsal OB strongly project to amygdala and mediate innate odor responses, whereas ventral OB accounts for processing of learned odorants [37]. To assess whether distinct activity patterns entrain the dorsal versus ventral OB at neonatal age, we compared RR and theta bursts from both subdivisions (S3 Fig). The power of RR was similar in both subdivisions (dorsal: median 233.12 μV^2^, iqr 153.42–418.09, *n* = 7; ventral: median 335.75 μV^2^, iqr 195.91–452.52, *n* = 10; *p* = 0.54, Wilcoxon rank-sum test, S3C Fig). Similarly, theta burst occurrence (dorsal: median 4.65 bursts/min, iqr 3.87–5.55; ventral: median 5.09 bursts/min, iqr 4.27–5.30, *p* = 0.74, Wilcoxon rank-sum test), duration (dorsal: median 6.76 s, iqr 4.44–8.86 s; ventral: median 3.54 s, iqr 1.65–5.01 s; *p* = 0.09, Wilcoxon rank-sum test), amplitude (dorsal: median 73.80 μV, iqr 59.56–75.37; ventral: median 66.59 μV, iqr 59.10–72.37; *p* = 0.67, Wilcoxon rank-sum test, S3D Fig), and relative power (dorsal: median 493.98, iqr 430.71–763.00; ventral: median 452.63, iqr 395.3–1,071.6, *p* = 0.96, Wilcoxon rank-sum test, S3C Fig) were comparable between OB subdivisions. These data indicate that the dorsal and ventral OB show similar activity at early postnatal age. Except when otherwise indicated, further investigation focused on the ventral OB subdivision, taking into account its role for learning processes in relation with the limbic system [37].

Coordinated patterns in the sensory periphery have been reported to critically depend on the brain state, diminishing or even disappearing in the presence of anesthetics [38,39]. In contrast, early oscillations in the developing brain have often been investigated in the presence of urethane anesthesia [9,35,40,41]. Rodent pups spend most of the time sleeping. The sleep-mimicking action of urethane might explain the similar patterns of neuronal activity previously observed in anesthetized and sleeping rodent pups [14,42]. To assess the influence of urethane on RR and theta bursts, we recorded from both ventral (*n* = 12) and dorsal OB (*n* = 6) of neonatal mice before and after urethane injection. Anesthesia did not change the overall structure of OB activity, with continuous RR and discontinuous theta bursts persisting (S4A Fig and S1 Table). Both the power of RR and the occurrence of theta bursts remained unchanged (S4B Fig). However, urethane anesthesia profoundly reduced theta burst duration (S4B Fig), augmenting those time windows lacking theta band activity and therefore the fragmented appearance of neonatal activity in OB (S4B Fig).

These data indicate that, independent of OB subdivision and brain state, the neonatal OB shows two main patterns of early oscillatory activity: continuous RR activity and discontinuous theta bursts.

### Mechanisms underlying the generation of continuous and discontinuous oscillatory activity in the neonatal OB

To elucidate the mechanisms contributing to the generation of continuous RR and discontinuous theta bursts in the OB of neonatal mice, we used two experimental approaches. First, the temporal coupling between respiration and continuous 2–4 Hz oscillations in OB suggests that nasal air flow contributes to RR generation. To test this hypothesis, we reduced the nasal air flow by unilateral naris occlusion with silicon adhesive in P8–10 pups (*n* = 12). MUA and oscillatory activity of OB were recorded before and after naris occlusion. While unilateral deprivation did not change the overall structure of OB activity patterns, it reduced the RR power from 396.05 μV^2^ to 293.30 μV^2^ (baseline: iqr 232.58–570.88 μV^2^; occlusion: iqr 136.10–410.14 μV^2^, *p* = 0.0009, Wilcoxon signed-rank test). By contrast, the theta bursts in OB were not affected by naris occlusion (baseline: median: 643.45 μV^2^, iqr 342.6–1,009.7; occlusion: median 700.35 μV^2^, iqr 284.5–1,240.8; *p* = 0.91, Wilcoxon signed-rank test). Correspondingly, the firing rate during RR (baseline: median 1.18 Hz, iqr 0.26–2.45) as well as coupling strength (i.e., PPC) between units and RR (baseline: median 8.50 × 10^−4^, iqr 0–0.0084, 1 outlier removed) decreased after naris occlusion (firing rate: occlusion: median 0.75 Hz, iqr 0.25–1.91, *p* = 0.021, Wilcoxon signed-rank test; spike-LFP coupling strength: occlusion: median −3.09 × 10^−5^, iqr −2.76 × 10^−4^ to 1.56 × 10^−4^; *p* = 0.049, Wilcoxon signed-rank test, 1 outlier removed). The temporal structure of OB firing in relationship to theta bursts remained unchanged after naris occlusion (spike-LFP coupling strength for baseline: median 2.09 × 10^−4^, iqr −0.0001 to 0.0015; occlusion: median −1.19 × 10^−4^, iqr −3.54 × 10^−4^ to 1.59 × 10^−4^; *p* = 0.19, Wilcoxon signed-rank test, 1 outlier removed). Thus, RR activity, but not theta bursts, critically depends on nasal air flow.

**Fig 3:**
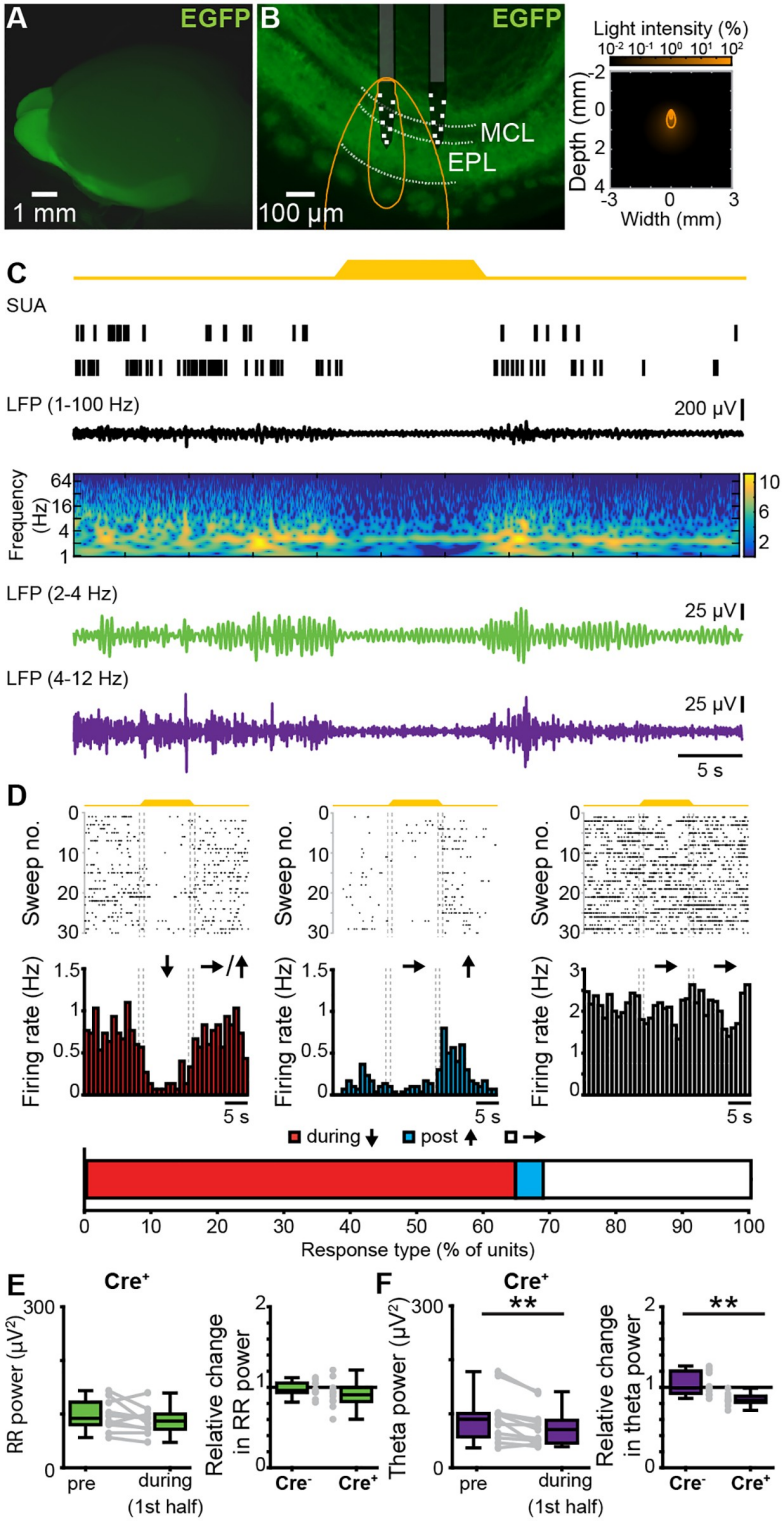
Effects of optogenetic silencing of MTCs on the patterns of oscillatory activity in the neonatal OB. (A) Photograph of the brain of a P8 cre^+^ Tbet-cre;ArchT-EGFP mouse (left) showing EGFP-fluorescent MTCs cell bodies and their projections. (B) Left, photograph of a 100 μm–thick coronal section including the OB from a P8 cre^+^ Tbet-cre;ArchT-EGFP mouse. The position of recording sites in MCL and EPL layers is marked by white squares. The light guide ending just above the recording sites is shown in gray. The iso-contour lines of light spreading calculated using Monte Carlo simulation are shown in yellow. Right, propagation of light intensity in the brain as predicted by Monte Carlo simulation. Yellow lines correspond to the iso-contour lines for light power of 1 and 10 mW/mm^2^, respectively. (C) Neuronal firing (SUA) and LFP band-pass filtered for different frequency bands (broad 1–100 Hz, RR 2–4 Hz, theta 4–12 Hz) in response to light (yellow, 594 nm) stimulation of MTCs in a P8 cre^+^ Tbet-cre;ArchT-EGFP mouse. Traces are accompanied by the color-coded wavelet spectrogram of LFP shown at an identical timescale. (D) Raster plots and peristimulus time histograms displaying the firing of MTCs in response to light stimulation. The color-coded bar (bottom) displays the fraction of cells that responded with a firing decrease during stimulus (red), constant firing during stimulus but a firing increase post stimulus (blue), and unchanged firing rate (white). (E) Box plots displaying the absolute power before and during light stimulation in cre^+^ pups (left) and the relative change of RR activity in neonatal OB of cre^+^ and cre^−^ mice (right). Gray dots and lines correspond to individual animals. (F) Same as E for discontinuous theta bursts (**p < 0.01, left: signed-rank test, right: rank-sum test). Data are available in S1 Data. EGFP, enhanced green fluorescent protein; EPL, external plexiform layer; LFP, local field potential; MCL, mitral cell layer; MTC, mitral and tufted cell; OB, olfactory bulb; P, postnatal day; RR, respiration-related rhythm; SUA, single-unit activity.

The second experimental approach aimed at assessing the role of MTCs, the OB projection neurons, to the generation of coordinated patterns of oscillatory activity. For this, we selectively manipulated MTC firing by light in P8–10 pups bred from crossing hemizygous Tbet-cre mice with R26-homozygous R26-ArchT-EGFP mice. By these means, MTCs of Cre^+^ mice selectively expressed the proton pump ArchT fused with enhanced green fluorescent protein (EGFP). Already at P8, the fusion protein expression was robust both in MTC somata (S5A Fig) and axonal projections targeting the posterior part of the cerebrum (Fig 3A). Cre^−^ mice were used as controls.

In a first experiment, we tested the efficiency of light-dependent MTC silencing in neonatal OB by performing whole-cell patch-clamp recordings from biocytin-filled EGFP-positive neurons (*n* = 7 cells) in coronal slices containing the OB of P8–10 R26-heterozygous Tbet-cre;R26-ArchT-EGFP mice (*n* = 5) (S5A Fig). Yellow light pulses (595 nm, 5 s, 0.2–0.6 mW) triggered MC hyperpolarization from −49.96 mV to −58.39 mV (baseline: iqr −57.28 to 45.61 mV; light administration: iqr −63.10 to 48.89 mV, Wilcoxon signed-rank test, *p* = 0.0078) and, consequently, inhibition of firing (S5B Fig). Since MTCs are strongly interconnected within local circuits, we tested whether light pulses caused MTC silencing also in the presence of synaptic inputs. To mimic such inputs, we paired the light stimulation with depolarizing current pulses of different intensities. Upon injections ≤60 pA, light stimulation still efficiently blocked action potential discharge in ArchT-EGFP-expressing MCs (S5C Fig).

Next, we assessed the contribution of MTC firing to the patterns of oscillatory activity in OB by performing extracellular recordings of LFP and MUA in OB of P8–10 R26-heterozygous Cre^+^ (*n* = 12) and Cre^−^ (*n* = 11) Tbet-cre;ArchT-EGFP mice in vivo. Upon light stimulation, the majority (64.58%, 31/48) of MTCs responded with a pronounced firing rate decrease from a median of 1.2 Hz (iqr 0.66–2.26) before to 0.45 Hz (iqr 0.13–0.99) during light exposure. None of the units augmented the firing during illumination, and only a few units (4.14%, 2/48) showed a post-stimulus firing increase (Fig 3D). Some units (31.25%, 15/48), most likely non-MTCs located close to the MCL, did not respond to light stimulation. Local silencing of MTCs modified the coordinated activity of OB. The properties of RR and theta bursts (theta burst power: *p* = 0.23, Wilcoxon rank-sum test) were largely similar in Cre^+^ and Cre^−^ mice under baseline conditions (i.e., before light stimulation). Only the power of RR activity was slightly different (RR power: *p* = 0.03, Wilcoxon rank-sum test). Upon light stimulation, the RR power in Cre^+^ pups did not change (prestimulus: median 92.27 μV^2^, iqr 80.12–122.36; during stimulus: median 86.99 μV^2^, iqr 71.96–100.06, *p* = 0.2324, Wilcoxon signed-rank test, 2 outliers removed, Fig 3E). In contrast, theta power in Cre^+^ pups significantly decreased during light stimulation (prestimulus: median 89.73 μV^2^, iqr 57.28–100.73; during stimulus: median 70.58 μV^2^, iqr 45.70–87.91, *p* = 0.0049, Wilcoxon signed-rank test, 1 outlier removed, Fig 3F). The theta responses to light differed between Cre^+^ (median 0.84 μV^2^, iqr 0.81–0.89) and Cre^−^ pups (median 0.99 μV^2^, iqr 0.93–1.20, *p* = 0.0024, Wilcoxon rank-sum test, 3 outliers from expression group removed), whereas RR during light stimulus was similar in the two groups (cre^+^, median 0.91, iqr 0.82–1.0; cre^−^ pups median 0.96, iqr 0.93–1.05, *p* = 0.3447, Wilcoxon rank-sum test, 1 outlier from control group, 2 outliers from expression group removed) (Fig 3E and 3F).

To assess whether MTCs contribute to theta burst generation through intrinsic membrane properties or as result of interactions within local circuits, we performed whole-cell patch-clamp recordings from visually identified MCs in vitro. Analysis of power distribution of subthreshold membrane oscillations revealed no peak within theta frequency band. In line with previous findings from adult cortical structures (e.g., LEC [43]), MCs showed membrane oscillations between 2 and 4 Hz (S6 Fig). These data indicate that MTCs contribute to neonatal theta oscillations through interactions within local circuits.

Taken together, these data show that RR and theta bursts in the neonatal OB have different origin. Whereas RR critically depends on nasal air flow, MTC activity within local circuits is necessary for the entrainment of OB in theta bursts.

### Theta bursts in OB drive discontinuous oscillations and time the firing in the neonatal LEC

The presence of both direct axonal MC-to-LEC projections and early patterns of oscillatory activity in OB led to the question of their relevance for the emergence of functional assemblies in the neonatal LEC. In contrast to the documented relevance of entorhinal output for developing limbic circuits [11], the role of sensory inputs for the functional maturation of LEC is still unknown.

**Fig 4:**
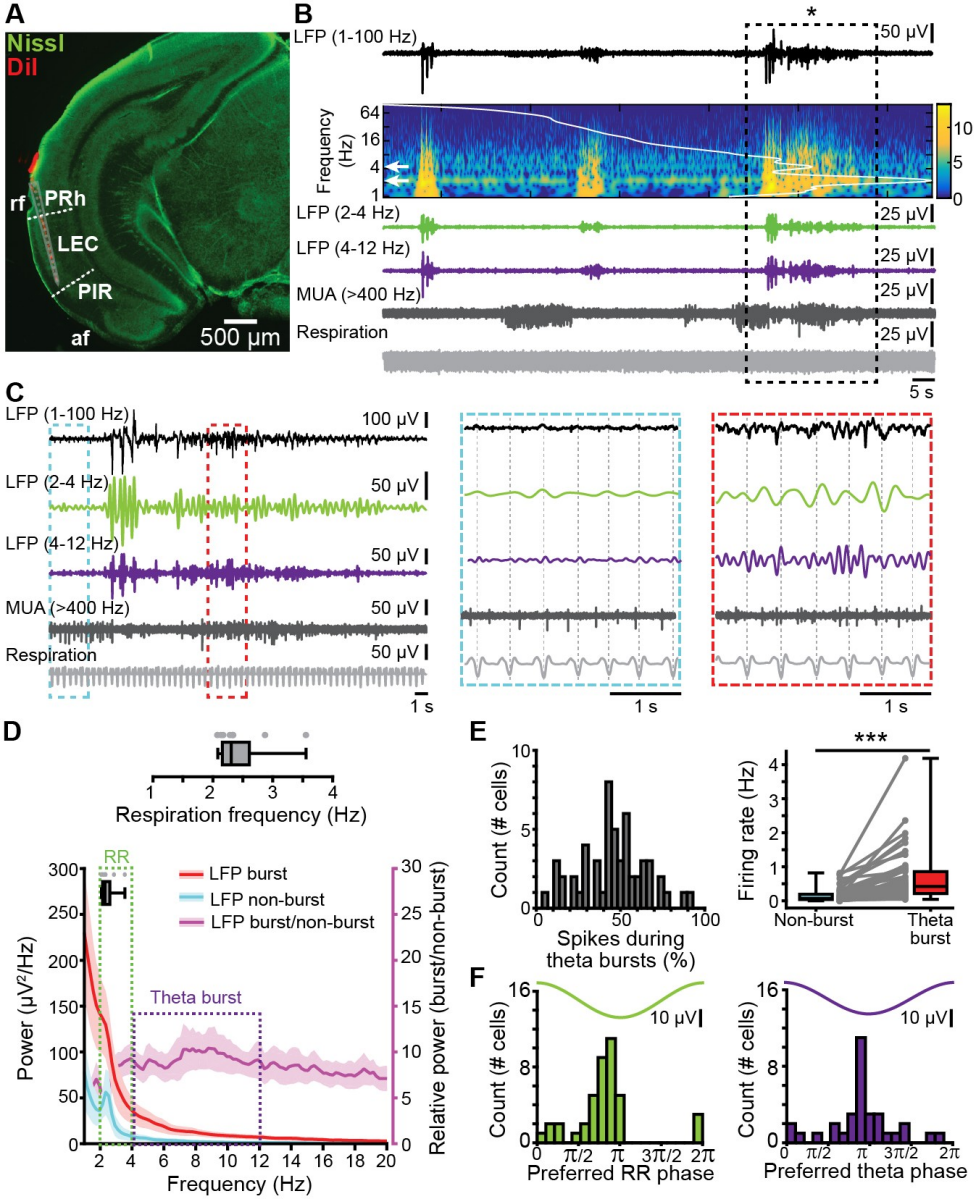
Continuous and discontinuous patterns of oscillatory activity in the neonatal LEC. (A) Digital photomontage reconstructing the track of the DiI-labeled multisite recording electrode (red) in a Nissl-stained (green) 100 μm–thick coronal section including LEC from a P9 mouse. The gray dots show the position of the 16 recording sites. (B) LFP recording of the oscillatory activity in LEC of a P10 mouse displayed band-pass filtered in different frequency bands and accompanied by the wavelet spectrogram (white line represents time-averaged power of the trace) as well as simultaneously recorded MUA (high-pass filter > 400 Hz) and respiration. (C) Characteristic slow continuous oscillatory activity and theta bursts from the trace shown in B when displayed at higher magnification. (D) Power spectra (mean ± SEM) of LFP in LEC during nonburst activity (blue) and discontinuous bursts (red) as well as of theta bursts normalized to nonbursting activity (purple). The respiration frequency was depicted as a horizontal bar and expanded at a larger scale (top). (E) Temporal relationship between neuronal firing and network oscillations in LEC. Left, histogram showing the percentage of spikes occurring during theta burst for all clustered units. Right, box plot depicting the firing rates of LEC units during nonburst periods and theta burst periods. Gray dots and lines correspond to individual cells (Wilcoxon signed-rank test, ***p < 0.001). (F) Histograms depicting the phase locking of LEC neurons to RR (left) and theta activity (right). Only significantly locked cells were used for analysis. Data are available in S1 Data. af, amygdaloid fissure; LEC, lateral entorhinal cortex; LFP, local field potential; MUA, multiunit activity; P, postnatal day; PIR, piriform cortex; PRh, perirhinal cortex; rf, rhinal fissure; RR, respiration-related rhythm.

Multisite extracellular recordings of LFP and MUA from the layer II/III of LEC from P8–10 mice in vivo (*n* = 11) (Fig 4A) confirmed the previously reported presence of discontinuous theta bursts with large amplitude (median 154.14 μV, iqr 101.10–191.65) and a duration of 5.15 s (iqr 4.13–8.48) (Fig 4B–4D). They appear superimposed on a slow rhythm (2–4 Hz) that continuously entrains the neonatal LEC and has been overlooked in previous investigations. This slow pattern of activity that was present both during theta bursts (median area power 526.25 μV^2^, iqr 307.68–1,171.85) and “silent” periods (median area power 86.57 μV^2^, iqr 52.55–344.43) temporally correlated with the simultaneously recorded respiration and was therefore classified as entorhinal RR. These results demonstrate that the respiration-entrained brain rhythms [44], a powerful mechanism of long-range coupling [45], emerge early during development. Beside oscillatory patterns, neonatal LEC generates prominent firing concentrated during theta bursts (median 0.42 Hz, iqr 0.22–0.86 versus nonbursting periods median 0.07 Hz, iqr 0.04–0.19, *p* = 1.72 × 10^−10^, Wilcoxon signed-rank test, *n* = 54 cells from 11 mice) (Fig 4E). We next assessed the coupling strength between firing and oscillatory activity. Similar fractions of entorhinal neurons were phase-locked to RR (75.93%, 41/54 units) and theta bursts (61.11%, 33/54 units, *p* = 0.1, χ^2^(1) = 2.7472). The strength of coupling assessed by PPC was also stronger for RR (median: 0.208, iqr 0.202–0.217) than for theta (median: 0.205, iqr 0.196–0.211, *p* = 2.98 × 10^−4^, Wilcoxon rank-sum test, 4 outliers removed, *n* = 50 units), with most cells being locked to the trough of RR and theta oscillation (Fig 4F).

**Fig 5:**
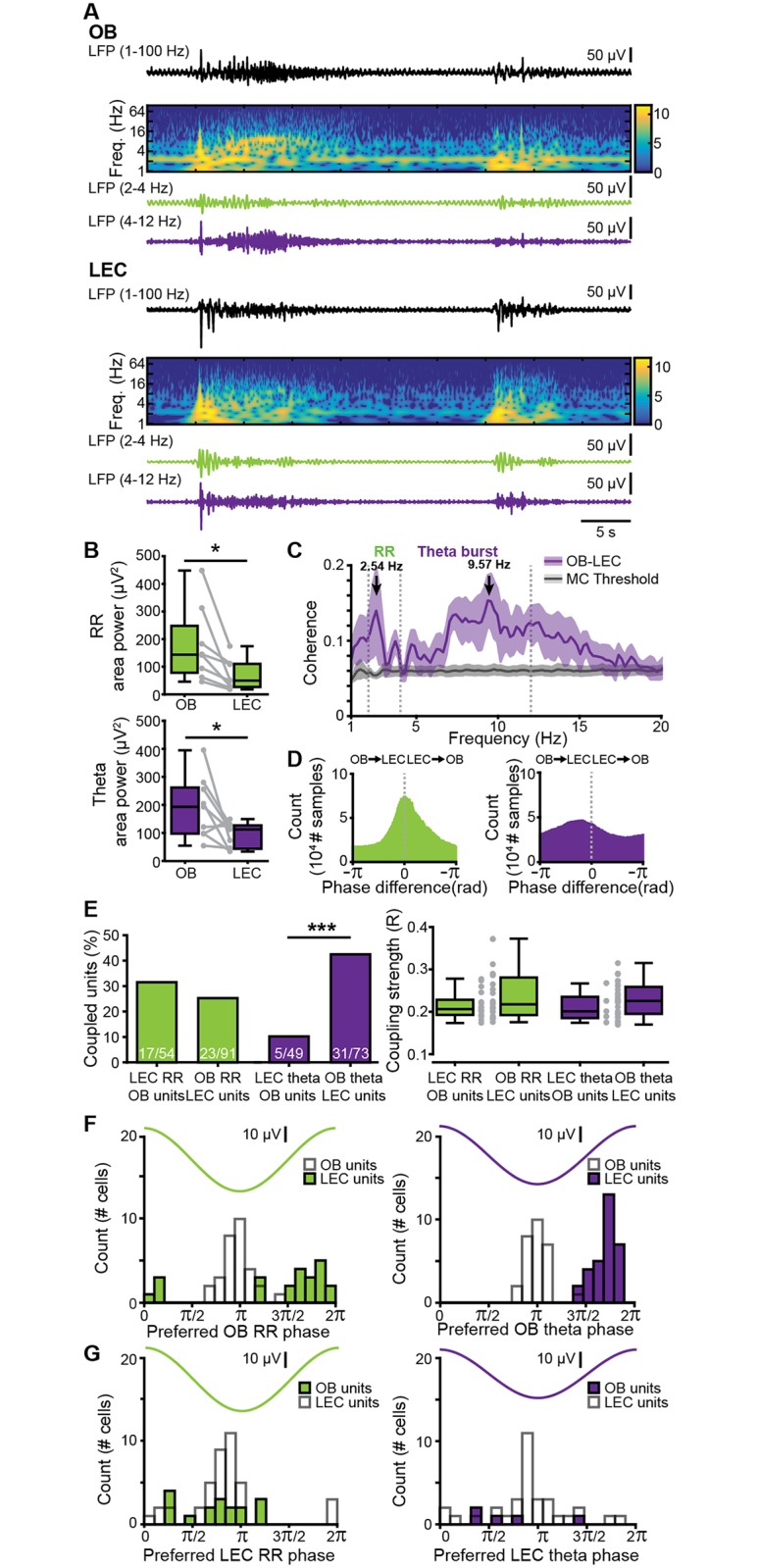
Frequency-dependent functional coupling between neonatal OB and LEC. (A) Characteristic traces of band-pass-filtered LFP recorded simultaneously in OB (top) and LEC (bottom) of a P9 mouse, displayed together with wavelet spectrograms showing the frequency (“Freq.”) content. Note the temporal correlation between discontinuous theta bursts in both areas. (B) Box plots displaying RR power (top, green) and theta burst power (bottom, purple) in OB and LEC. Gray lines and dots correspond to individual pups. (*p < 0.05, Wilcoxon signed-rank test). (C) Plot of imaginary part of coherence between OB and LEC showing prominent peaks in RR and theta band. The gray line corresponds to the significance threshold as assessed by Monte Carlo simulation. (D) Histograms of phase differences between RR (left, green) and theta (right, purple) activity recorded simultaneously in OB and LEC. (E) Left, bar diagram displaying the percentage of OB units coupled to the RR (green) and theta bursts (purple) in LEC and the percentage of LEC units coupled to the RR (green) and theta bursts (purple) in OB. Right, box plot showing the coupling strength of OB cells significantly locked to LEC oscillations (green: RR, purple: theta bursts) and of LEC cells significantly locked to OB oscillations (green: RR, purple: theta bursts). Gray dots correspond to individual cells (χ^2^ test of proportions, ***p < 0.001). (F) Histograms showing the distribution of preferred phases of LEC cells significantly locked to RR (left) and OB theta bursts (right) in neonatal OB. For comparison, histograms of OB cells locked to the respective OB rhythm are plotted as white bars. (G) Histograms showing the distribution of preferred phases of OB cells significantly locked to RR (left) and theta bursts (right) in neonatal LEC. For comparison, histograms of LEC cells locked to the respective LEC rhythm are plotted as white bars. Data are available in S1 Data. LEC, lateral entorhinal cortex; LFP, local field potential; MC, mitral cell; OB, olfactory bulb; P, postnatal day; RR, respiration-related rhythm.

Simultaneous recordings from OB and LEC (*n* = 9) of neonatal mice gave first insights into their dynamic coupling (Fig 5A). Although both areas showed similar oscillatory activity, their power significantly differed. Both RR power (OB: median 143.62 μV^2^, iqr 78.03–247.78; LEC: median 109.91, iqr 26.72–110.51, *p* = 0.0499, Wilcoxon signed-rank test, 1 outlier removed) and theta power (OB: median 193.39 μV^2^, iqr 97.47–262.01, LEC: median 112.37 μV^2^, iqr 43.38–127.36, *p* = 0.0273, Wilcoxon signed-rank test) were higher in OB as compared to LEC (Fig 5B). Analysis of the temporal correspondence of theta bursts in OB and LEC revealed that 48.70% of them co-occurred with more than 60% temporal overlap. The coupling strength assessed by imaginary spectral coherence, which excludes synchrony effects due to volume conductance [46], revealed that the OB–LEC coupling is evident in both slow frequencies (i.e., RR) and theta band (i.e., theta bursts) (Fig 5C). In line with anatomical data, we detected no differences in the coupling of dorsal and ventral OB with LEC. Both relative occurrence of co-occurring events (dorsal: median 27.04%, iqr 20.08–33.98%; ventral: median 21.83%, iqr 15.66–35.14%; *p* = 0.67, Wilcoxon rank-sum test) and mean imaginary coherence in both RR (dorsal: median 0.11 Hz, iqr 0.09–0.14 Hz; ventral: median 0.08 Hz, iqr 0.05–0.10 Hz; *p* = 0.13, Wilcoxon rank-sum test) and theta frequency range (dorsal: median 0.07 Hz, iqr 0.06–0.11 Hz; ventral: median 0.06 Hz, iqr 0.06–0.09 Hz; *p* = 0.54, Wilcoxon rank-sum test) were similar for dorsal and ventral OB in relationship to LEC (S3 Fig). These data are in line with anatomical investigations in adult mice [47] as well as with our tracing data (Fig 1), showing that FG injection into neonatal LEC leads to homogenous MTC labeling throughout the OB.

To assess the influence of anesthesia on entorhinal activity patterns and coupling between LEC and OB, we recorded both areas in mouse pups before and after urethane intraperitoneal (i.p.) injection (*n* = 18). Urethane did not change the overall spectral distribution of activity patterns in LEC. As in the nonanesthetized state, RR and theta bursts were the main patterns of entorhinal activity, yet the RR power decreased and theta power augmented under urethane action (S7A Fig and S1 Table). Urethane affected the duration of theta bursts and slightly increased their occurrence (S7B Fig). The synchrony between OB and LEC varied in magnitude but not frequency distribution. The imaginary coherence peaked at 2–4 Hz and at 5–20 Hz, corresponding to RR and theta–beta frequencies, respectively. Whereas mean RR coherence did not differ between states (*p* = 0.17, Wilcoxon rank-sum test, 2 outliers removed), theta coherence was higher in the presence of urethane (*p* = 0.0034).

These data indicate that, independent of brain state and anatomical subdivision, OB and LEC are tightly coupled at neonatal age both being synchronized in continuous RR and discontinuous theta oscillations.

Since feedforward projections from MCs to LEC are dense, whereas feedback projections from LEC to OB are rather sparse, we asked whether the functional coupling between the two areas is directed and, if so, whether directionality is frequency specific. To estimate the directionality of OB–LEC coupling, we used two approaches. First, we assessed the phase lag between LFP in OB and LEC. Although the phase lag for continuous RR was centered to 0, it peaked in negative range for theta bursts, indicating that OB theta bursts most likely drive LEC theta oscillations (Fig 5D). Second, we analyzed the temporal relationship between spiking activity in one area and either LFP or spiking in the other area. For RR, a similar number of clustered units in OB and LEC were phase-locked to RR in LEC (31.48%, 17/54) and OB (25.27%, 23/91, *p* = 0.54, χ^2^(1) = 0.38, χ^2^ test of proportions), respectively, and their coupling strengths were comparable (OB cells to LEC RR: median 0.21, iqr 0.19–0.23; LEC cells to OB RR: median 0.22, iqr 0.19–0.28, *p* = 0.35, Wilcoxon rank-sum test, Fig 5E). In contrast, a significantly higher fraction of LEC neurons were phase-locked to theta bursts in OB (42.47%, 31/73) when compared to OB neurons timed by entorhinal theta phase (10.20%, 5/49, *p* = 1.28 × 10^−4^, χ^2^(1) = 14.67, χ^2^ test of proportions, Fig 5E). The coupling strengths of these neuronal populations, however, were comparable (OB cells to LEC theta: median: 0.22, iqr 0.19–0.24; LEC cells to OB theta: median: 0.23, iqr 0.20–0.26, *p* = 0.44, Wilcoxon rank-sum test, 1 outlier removed from OB theta–LEC units group). MTCs preferentially fire during the trough of RR activity and theta bursts in OB, whereas LEC cells preferentially fire on the rising phase after the trough of OB rhythms, indicating that MTC firing precedes LEC cell firing by about a third of a cycle (Fig 5F and 5G).

Together, these data suggest that the continuous RR rhythm is not involved in directed information flow within OB–LEC circuits, whereas theta bursts in OB drive the oscillatory entrainment of LEC.

### Pharmacological blockade of OB firing diminishes the slow and fast oscillatory activity in OB–LEC circuits

**Fig 6:**
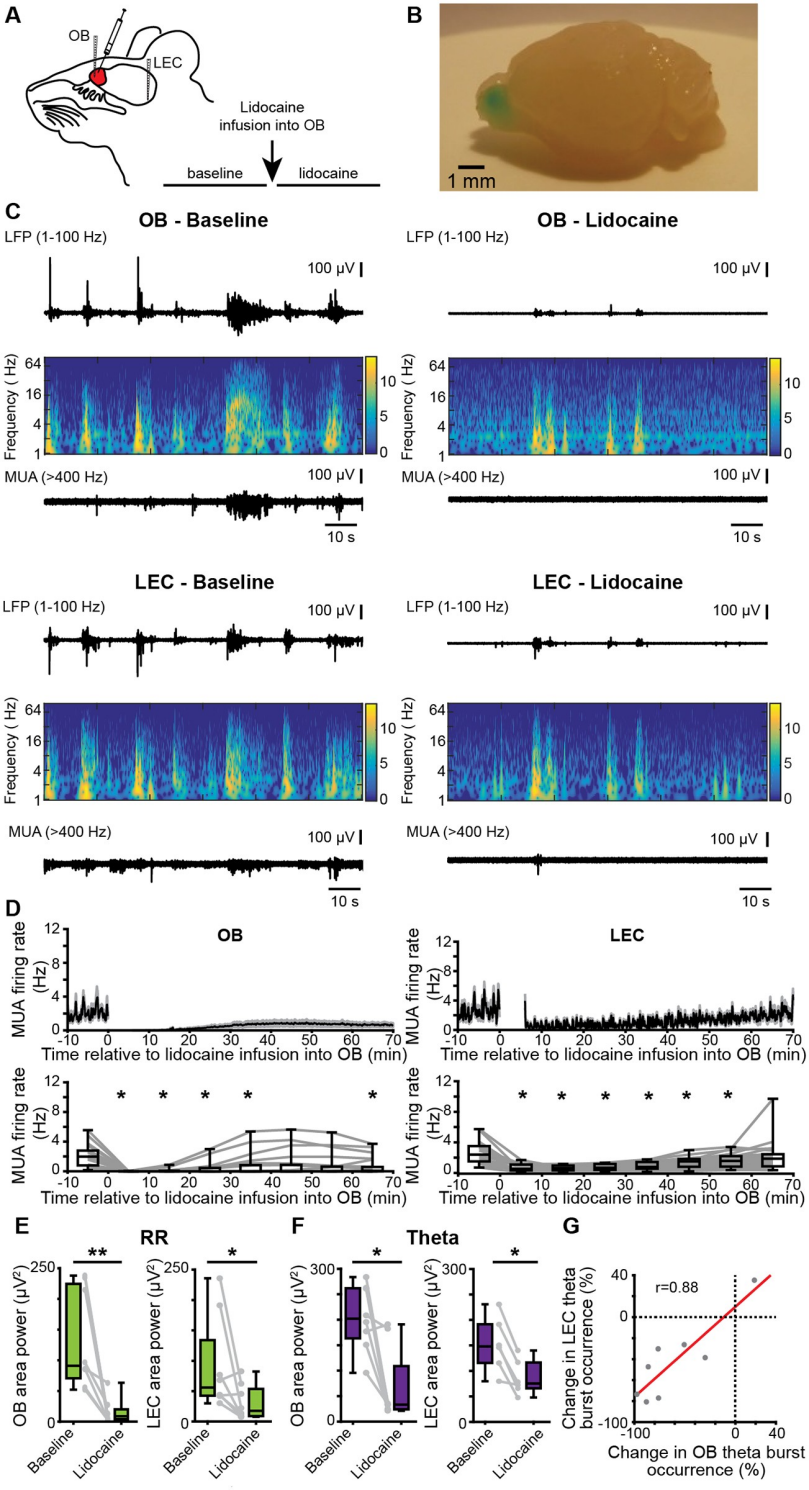
Effects of pharmacological blockade of neuronal firing in OB on patterns of oscillatory activity in OB–LEC circuits. (A) Schematic drawing of experimental protocol. (B) Photograph of the brain of a P10 mouse showing the confinement of injections to one hemisphere of the OB. For visualization, the same volume of methylene blue was used. (C) Characteristic LFP traces (black, filtered 1–100 Hz) recorded in OB (top) and LEC (bottom) of a P9 mouse before (left) and after (right) lidocaine infusion, displayed together with the wavelet spectrograms of the LFP and simultaneously recorded MUA. (D) Top, mean MUA firing rate in OB (left) and LEC (right) before and after lidocaine infusion. The time of infusion is considered 0. Bottom, box plots displaying the mean MUA in OB (left) and LEC (right) before and after lidocaine infusion (Friedmann test, Wilxocon signed-rank test with Bonferroni correction for post hoc comparison, *p < 0.0071). (E) Box plots displaying the power of RR activity in OB and LEC in the RR band before and after lidocaine infusion. Gray dots and lines correspond to individual animals (Wilcoxon signed-rank test, *p < 0.05; **p < 0.01). (F) Same as E for the theta burst activity in neonatal OB and LEC. (G) Scatterplot displaying the relationship between the occurrence changes (percent of baseline) of OB and LEC theta bursts (r = 0.008, *p* = 0.0039, Pearson correlation). Data are available in S1 Data. LEC, lateral entorhinal cortex; LFP, local field potential; MUA, multiunit activity; OB, olfactory bulb; P, postnatal day; RR, respiration-related rhythm.

To confirm that OB theta bursts are necessary for the generation of LEC theta bursts, we pharmacologically abolished the neuronal activity by unilateral pressure injection of the voltage-dependent sodium channel (hence action potential) blocker lidocaine (4% in sterile saline) into OB. Extracellular recordings of LFP and MUA were performed simultaneously from OB and LEC of mice (*n* = 8) before and after lidocaine injection in vivo (Fig 6A). The injected lidocaine volume of 4 μl was proven to not spread across the borders of OB (Fig 6B). Lidocaine abolished OB firing within 10 min of injection from a median baseline firing rate of 1.97 Hz (iqr 0.77–2.80) to 0.00 Hz (iqr 0.00–0.02). A partial recovery was observed after 30–40 min (χ^2^(7) = 45.04, *p* = 1.34 × 10^−7^, Friedman test, with Wilcoxon signed-rank post hoc test with Bonferroni correction) (Fig 6D). The firing of entorhinal neurons was also significantly reduced after lidocaine treatment in OB from a median baseline firing rate of 2.4 Hz (iqr 1.46–3.60) to 0.52 Hz (iqr 0.32–1.09) within the first 10 min after injection (χ^2^(7) = 135.50, *p* = 4.45 × 10^−26^, Friedman test, with Wilcoxon signed-rank post hoc test with Bonferroni correction). The decrease of firing rates in both areas was accompanied by changes of oscillatory network activity. In OB, the power of RR (baseline: median 91.05 μV^2^; iqr 70.66–224.40; lidocaine: median 8.88, iqr 3.90–20.16; *p* = 0.0078, Wilcoxon signed-rank test) as well as the occurrence (baseline: median 4.76 bursts/min, iqr 3.58–5.93; lidocaine: 1.03 bursts/min, iqr 0.76–1.56, *p* = 0.0234, Wilcoxon signed-rank test), duration (baseline: median 4.41 s, iqr 3.78–4.77; lidocaine: median 2.35, iqr 1.89–2.88, *p* = 0.0078, Wilcoxon signed-rank test), and power (baseline: median 202.07 μV^2^, iqr 163.62–261.95; lidocaine: median 33.19, iqr 24.14–109.00, *p* = 0.0156, Wilcoxon signed-rank test) of theta bursts were reduced. In LEC, the power of RR (baseline: median 55.88 μV^2^, iqr 42.48–134.05; lidocaine: median 17.71, iqr 9.07–53.80, *p* = 0.0391, Wilcoxon signed-rank test) as well as the duration (baseline: median 4.41 s, iqr 3.74–4.58; lidocaine: median 3.24, iqr 3.14–3.56, *p* = 0.0156, Wilcoxon signed-rank test, 1 outlier removed) and power (baseline: median 148.11 μV^2^, iqr 115.76–191.62; lidocaine: median 75.50, iqr 65.99–116.63, *p* = 0.0313, Wilcoxon signed-rank test, 2 outliers removed) of theta bursts were decreased after blockade of OB firing. Moreover, the lidocaine-induced changes in theta burst occurrence were highly correlated between OB and LEC (r = 0.88, *p* = 0.0039, Pearson correlation, Fig 6G).

These data indicate that blocking of neuronal firing in OB causes massive diminishment of coordinated activity in both OB and LEC.

### Optogenetically evoked OB theta bursts lead to neuronal firing and network oscillations in neonatal LEC

**Fig 7:**
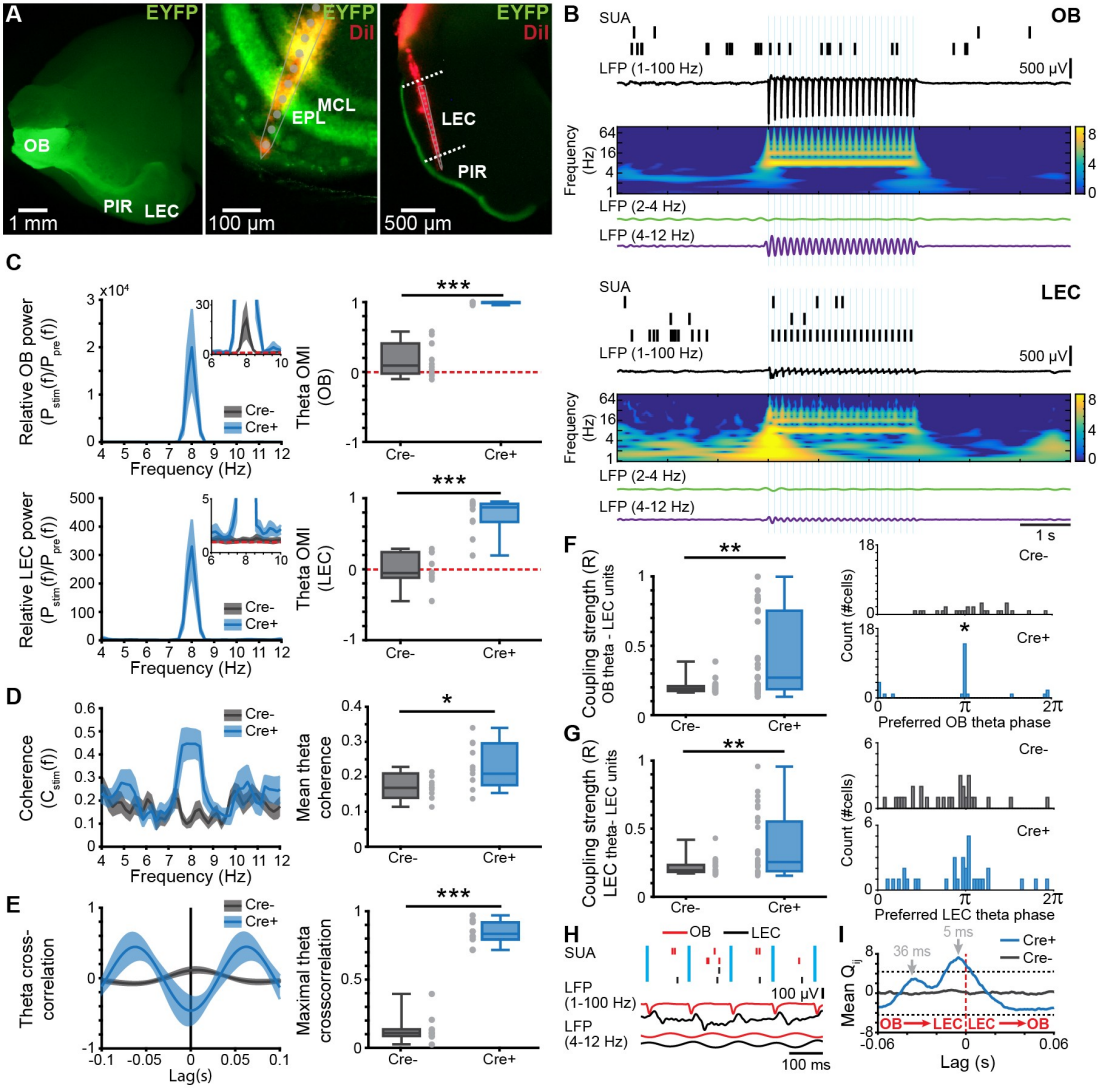
Effects of rhythmic optogenetic MTC activation on LEC oscillatory activity and single-unit entrainment. (A) Left, photograph of the ventral side of a brain from a P8 Cre^+^ Tbet-cre mouse showing EYFP-fluorescent MTC bodies in OB and their projections reaching PIR and LEC. Middle, photograph of the DiI-labeled optrode track into a 100 μm–thick coronal section of the OB from a P8 Cre^+^ Tbet-cre mouse. Right, photograph of the DiI-labeled electrode track into a 100 μm–thick coronal section of the LEC from a P8 Cre^+^ Tbet-cre mouse. (B) Spike trains from clustered units recorded simultaneously with the band-pass filtered (1–100 Hz, RR 2–4 Hz, theta 4–12 Hz) LFP in response to pulsed light (blue, 473 nm) stimulation of MTCs in OB (top) and LEC (bottom) of a P8 Cre^+^ Tbet-cre mouse. Traces are accompanied by the color-coded wavelet spectrograms of LFP shown at identical timescale. (C) Left, power spectra showing the relative LFP power change in OB (top) and LEC (bottom) after pulsed (8 Hz) light stimulation of MTCs in Cre^−^ and Cre^+^ mice. Inset, power spectra shown at higher magnification. Right, box plot showing OMI of theta power in OB (top) and LEC (bottom) of Cre^−^ and Cre^+^ mice (OB: *p* = 0.0002, LEC: *p* = 0.0004, Wilcoxon rank-sum test). For all plots, the red dotted line corresponds to unchanged power. (D) Left, plots of imaginary coherence between OB and LEC during pulsed (8 Hz) light stimulation of MTCs in Cre^−^ and Cre^+^ mice. Right, box plot displaying mean theta coherence during light stimulation of MTCs (*p* = 0.021, Wilcoxon rank-sum test). (E) Left, plots of cross-correlation between OB and LEC during light stimulation of MTCs in Cre^−^ and Cre^+^ mice. Right, box plot showing maximal cross-correlation during light stimulation of MTCs (*p* = 0.0002, Wilcoxon rank-sum test). (F) Left, coupling strength calculated as mean resultant vector length for LEC units to the OB theta rhythm during light stimulation of MTCs in Cre^−^ and Cre^+^ mice (*p* = 0.0055, Wilcoxon rank-sum test). Right, histograms showing the phase preference of LEC units (*p* = 0.01, Kuiper two-sample test). (G) Same as (F) for LEC units to LEC theta phase. (H) Spike trains in relationship to LFP in OB and LEC. Note the presence of both short and long delays between spikes from the two areas. (I) Mean standardized spike–spike cross-covariance of significant OB–LEC unit pairs from Cre^+^ mice (*n* = 27 pairs) and Cre^−^ mice (*n* = 61 pairs). Black dashed lines indicate the significance threshold. A negative time lag corresponds to OB → LEC. Data are available in S1 Data. EPL, external plexiform layer; EYFP, enhanced yellow fluorescent protein; LEC, lateral entorhinal cortex; LFP, local field potential; MC, mitral cell; MTC, mitral and tufted cell; OB, olfactory bulb; OMI, optogenetic modulation index; P, postnatal day; PIR, piriform cortex; RR, respiration-related rhythm; SUA, single-unit activity.

To prove the role of OB oscillatory activity for the entrainment of LEC, we transfected MTCs with a channelrhodopsin 2 (ChR2) mutant using a viral strategy. For this, we injected the construct pAAV-Ef1a-DIO hChR2(E123T/T159C)-EYFP (AAV9) unilaterally into the OB of Tbet-cre mice at P0–1 (*n* = 10 Cre^+^, *n* = 10 Cre^−^). At P8, hChR2-EYFP was expressed in OB MTCs (S8A Fig) and in the entire LOT, reaching LEC superficial layers (Fig 7A). In vitro whole-cell patch-clamp recordings from biocytin-filled enhanced yellow fluorescent protein (EYFP)-positive neurons (*n* = 4) confirmed that MCs were able to follow blue light pulses (470 nm, 3 ms duration, 0.77 mW) applied at frequencies between 2 and 32 Hz (S8B and S8C Fig).

Stimulation of MTCs with 3 ms–long light pulses at 8 Hz was performed simultaneously with LFP and MUA recordings from LEC in vivo. During baseline recordings (prior to light stimulation), RR power was comparable between Cre^−^ (controls) and Cre^+^ pups in OB (Cre^−^ pups: median: 485.50 μV2, iqr 339.81–778.70; Cre^+^ pups: median: 349.67 μV2, iqr 325.62–416.51, *p* = 0.32, Wilcoxon rank-sum test, 2 outliers removed) and LEC (Cre^−^ pups: median: 369.72 μV2, iqr 165.76–776.32; Cre^+^ pups: median: 214.78 μV2, iqr 156.66–248.04, *p* = 0.08, Wilcoxon rank-sum test, 1 outlier removed). Similarly, the power of theta bursts was comparable between Cre^−^ and Cre^+^ pups in OB (Cre^−^ pups: median: 333.25 μV2, iqr 168.13–537.81; Cre^+^ pups: median: 198.13 μV2, iqr 149.60–574.66, *p* = 0.68, Wilcoxon rank-sum test) and LEC (Cre^−^ pups: median: 327.24 μV2, iqr 143.33–503.64; Cre^+^ pups: median: 206.17 μV2, iqr 116.36–235.02, *p* = 0.21, Wilcoxon rank-sum test, 1 outlier removed). Light stimulation triggered MTC spiking shortly (3–5 ms) after stimulus onset as well as at longer delays (S9 Fig). Light stimulation evoked oscillatory activity peaking in the theta frequency band in both the OB and LEC of Cre^+^ pups (Fig 7B and 7C), whereas in Cre^−^, a weak light artifact was present in OB but not LEC (Fig 7C). The magnitude of light-evoked entorhinal activity was lower than that of OB oscillations. Correspondingly, the optogenetic modulation index (OMI) of theta band power in OB was significantly higher in Cre^+^ pups (median: 0.9964, iqr 0.9861–0.9979, *p* = 0.0002, Wilcoxon rank-sum test) when compared to Cre^−^ mice (median: 0.09, iqr −0.02 to 0.41). A similar augmentation of OMI was detected for LEC (Cre^+^: median: 0.87 iqr 0.67–0.92; Cre^−^: median: −0.05, iqr −0.12 to 0.24, *p* = 0.0004, Wilcoxon rank-sum test). Moreover, light stimulation increased the OB–LEC coupling by synchrony, measured by imaginary coherence in the theta band (Cre^+^ pups median: 0.22, iqr 0.19–0.30; Cre^−^ pups median: 0.17, iqr 0.15–0.18, *p* = 0.021, Wilcoxon rank-sum test) (Fig 7D) and maximal cross-correlation at the same frequencies (Cre^+^ pups: median: 0.83, iqr 0.79–0.92, Cre^−^ pups: median: 0.11, iqr 0.09–0.14, *p* = 0.0002, Wilcoxon rank-sum test) (Fig 7E). These results show that theta bursts generated through MTC activation in OB cause theta band entrainment in LEC of neonatal mice.

To investigate in more detail the coupling interactions between the two areas, we focused on the spiking activity of OB and LEC in relationship to the oscillatory phase and quantified both the number of phase-locked cells and the locking strength assessed by the mean resultant vector length. In Cre^−^ pups, 0% (0/28) of units from LEC were locked to the OB theta rhythm during light stimulation. In contrast, 31.25% (10/32) of units from the LEC of Cre^+^ mice were locked to the OB theta rhythm (*p* = 0.0012, χ^2^(1) = 10.5, χ^2^ test of proportions). The locking strength was significantly higher in Cre^+^ (median: 0.27, iqr 0.19–0.75) when compared to Cre^−^ mice (median: 0.19, iqr 0.17–0.21, *p* = 0.0055, Wilcoxon rank-sum test). Moreover, the distribution of preferred phases differed between Cre^−^ and Cre^+^ mice (*p* = 0.01, Kuiper two-sample test), with stronger locking of LEC units to the OB theta trough in Cre^+^ mice (Fig 7F). Next, the entrainment of LEC SUA by the evoked LEC theta rhythm was investigated. During the light stimulation period, 0% (0/28) of units from Cre^−^ mice were locked to theta, whereas 18.75% (6/32) units from LEC of Cre^+^ mice were significantly locked to LEC theta (*p* = 0.0157, χ^2^(1) = 5.8333, χ^2^ test of proportions). The locking strength of LEC units to LEC phase was significantly higher in Cre^+^ mice (median: 0.26, iqr 0.19–0.55) when compared to Cre^−^ mice (median: 0.19, iqr 0.17–0.23, *p* = 0.0013, Wilcoxon rank-sum test) (Fig 7G). However, the distribution of preferred LEC theta phases of LEC units during the light stimulation did not differ between the two groups of mice (*p* > 0.05, Kuiper two-sample test, Fig 7G).

To determine whether OB entrains LEC via mono- or polysynaptic connections, we isolated 51 single units recorded in the MCL of OB and calculated the standardized spike–spike cross-covariance between OB and LEC using previously developed algorithms [48]. Of a total of 202 unit pairs from Cre^+^ mice and 274 unit pairs from Cre^−^ mice (188 and 236 pairs, respectively) had sufficiently high firing rates (>0.05 Hz) and were used for further analysis. The mean standardized cross-covariance (Fig 7I) of all significant unit pairs (27 pairs from Cre^+^ mice, 61 pairs from Cre^−^ mice) peaked at −5 ms and −36 ms in Cre^+^ mice. A negative time lag corresponds to shifting LEC backwards in time; i.e., OB drives LEC. The first peak indicates monosynaptic connectivity, whereas the longer time lag of cross-covariance peak corresponds to polysynaptic connections, possibly involving the PIR, which has been reported to be tightly connected to both OB and LEC in adults [49]. These data indicate that MTC firing drives entorhinal activity via both mono- and polysynaptic projections. To investigate the role of neonatal PIR for OB–LEC coupling, we recorded simultaneously OB–LEC (*n* = 18 pups), OB–PIR (*n* = 7 pups), and LEC–PIR (*n* = 5 pups) and performed cross-covariance analysis of the corresponding spike train pairs. Previous studies showed that adult PIR receives strong innervation mostly from MCs, with tufted cells only targeting the ventrorostral part of the anterior PIR [16]. Our tracing experiments revealed that these projections are present already in neonatal mice (Fig 7A). Clustering of MUA led to identification of 61, 106, and 32 single units from OB, LEC, and PIR, respectively. Spike train pairs with firing rates >0.05 Hz (OB–LEC 251, OB–PIR 62, and LEC–PIR 142) were considered for analysis. Although spike trains from PIR and LEC were highly correlated, they lacked a preferred directionality as shown by the 0 ms lag of cross-covariance peak (S10A Fig, left plot). In contrast, OB–LEC and OB–PIR spike cross-covariance showed multiple peaks, yet they were of lower magnitude (S10A Fig, right plot). In line with the results of light stimulation of MTCs (Fig 7I), the first peak of OB–LEC cross-covariance had a negative 7 ms lag, indicating that monosynaptic projections from OB drive LEC firing. Additional peaks at longer delay reflect polysynaptic interactions corresponding to both OB → LEC and LEC → OB. OB–PIR spike cross-covariance peaked both at a shorter negative (i.e., most likely monosynaptic connection OB → PIR) and a longer positive (i.e., most likely polysynaptic connection PIR → OB) lag (S10A Fig). Quantification of mono- versus polysynaptic coupling among spike train pairs revealed that monosynaptic coupling occurred for 11% (28/251) of OB–LEC pairs and for 11% (7/62) of OB–PIR pairs, whereas polysynaptic coupling occurred for 8% (19/251) of OB–LEC and for 2% (1/62) of OB–PIR pairs. The highest extent of monosynaptic coupling has been observed for LEC–PIR pairs (68%, 97/142). These data indicate that in neonatal mice, OB drives LEC as well as PIR via monosynaptic projections with no direct functional feedback, despite the presence of sparse projections. Since more OB–LEC cell pairs were monosynaptically than polysynaptically coupled, and the spike-timing delay between PIR and LEC was 0 ms, we suggest that the indirect pathway from OB to LEC via PIR does not play a major role in the entrainment of neonatal activity.

### Odors boost the oscillatory activity in neonatal OB and LEC and augment their fast frequency coupling

**Fig 8:**
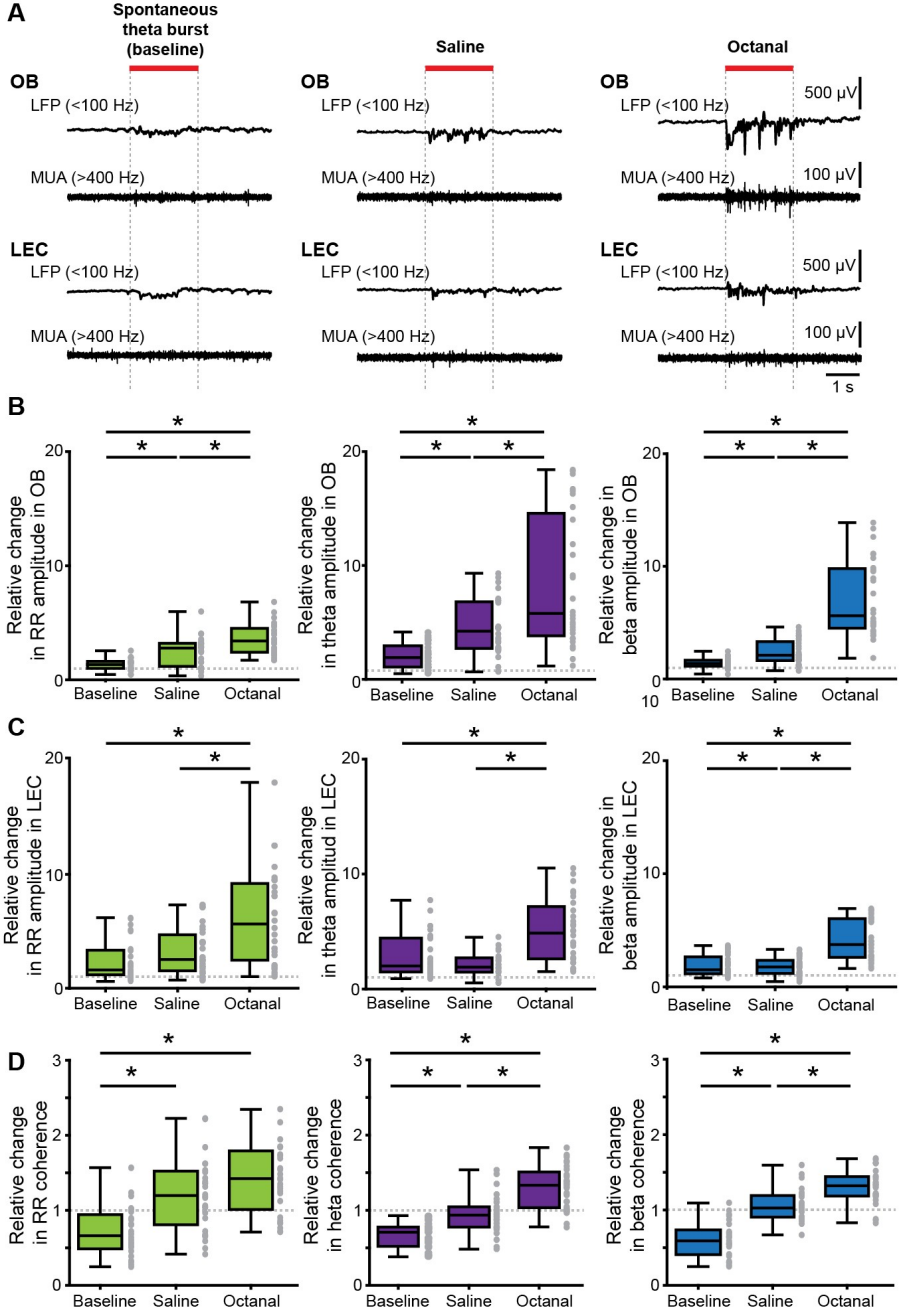
Odor-triggered activity patterns in OB and LEC of neonatal mouse. (A) Characteristic LFP traces (band-pass filtered 1–100 Hz) recorded in OB (top) and LEC (bottom) of a P9 mouse before (baseline, left) and after application of odors (saline, middle; octanal, right) displayed together with simultaneously recorded MUA. (B) Box plots showing odor-evoked changes in the amplitude of RR (left), theta (middle), and beta (right) activity in OB when normalized to baseline. (C) Same as B for LEC. (D) Box plots showing odor-evoked relative changes in OB–LEC coherence in RR (left), theta (middle), and beta (right) band when normalized to baseline. Gray dots correspond to individual trials (Kruskal-Wallis test, Wilcoxon rank-sum test with Bonferroni correction as post hoc test, *p < 0.0167). Data are available in S1 Data. LEC, lateral entorhinal cortex; MUA, multiunit activity; OB, olfactory bulb; P, postnatal day; RR, respiration-related rhythm.

In contrast to other sensory systems that lack peripheral sensitivity for environmental stimuli during early postnatal development, the olfactory system processes inputs already at birth. Therefore, the characterized coordinated patterns of oscillatory activity, RR, and theta bursts might have a dual origin, i.e., resulting from both spontaneous and/or stimulus-evoked activation of OB neurons. To gain first insights into the relevance of environmental stimuli on oscillatory activity and long-range entrainment of OB and LEC, we recorded brain activity evoked by odors inhaled via respiration of the anesthetized P8–10 mouse. Prominent oscillatory discharge with slow and fast frequencies and MUA were induced in OB by olfactometer-controlled exposure to odors, such as octanal (10%) (Fig 8A). We observed odor-evoked responses also in LEC, albeit at lower magnitude. Compared to theta bursts recorded in absence of stimuli (i.e., baseline) and to responses to saline, these octanal-evoked responses had a higher amplitude in RR frequency range both in OB (χ^2^ (2) = 36.05, *p* = 1.49 × 10^−8^, Kruskal-Wallis test, Wilcoxon rank-sum test with Bonferroni correction as post hoc test, removed outliers: baseline 2, saline 1) and LEC (χ^2^ (2) = 13.80, *p* = 0.001, Kruskal-Wallis test, Wilcoxon rank-sum test with Bonferroni correction as post hoc test, removed outliers: baseline 2, saline 1). Similarly, octanal augmented the amplitude of theta bursts in both regions (OB: χ^2^(2) = 36.30, *p* = 1.31 × 10^−8^, Kruskal-Wallis test, Wilcoxon rank-sum test with Bonferroni correction as post hoc test, LEC: χ^2^(2) = 20.52, *p* = 0.000035, Kruskal-Wallis test, Wilcoxon rank-sum test with Bonferroni correction as post hoc test, removed outliers: baseline 2, saline 1) (Fig 8B and 8C and Table 1). In contrast to coordinated theta burst activity recorded in the absence of olfactory stimulation, evoked responses included beta band (15–30 Hz) activity. Similar activity has been reported in the adult rodent olfactory system in Go/No-Go tasks [50,51], in response to predator odors [52,53], and in response to highly volatile odorants [54]. The amplitude of beta activity was significantly higher in the presence of octanal than during baseline or saline exposure both in OB (χ^2^(2) = 56.52, *p* = 5.33 × 10^−13^, Kruskal-Wallis test, Wilcoxon rank-sum test with Bonferroni correction as post hoc test, 1 outlier removed) and LEC (χ^2^(2) = 31.94, *p* = 1.16 × 10^−7^, Kruskal-Wallis test, Wilcoxon rank-sum test with Bonferroni correction as post hoc test) (Fig 8B and 8C and Table 1). The presence of odor-driven OB activity confirms the maturity of receptor cells and odor-processing mechanisms in the olfactory system at early postnatal age. Moreover, the presence of odor-driven LEC activity indicates that coordinated activity from OB drives the oscillatory entrainment of LEC. To determine which oscillatory patterns are mainly involved in these directed OB–LEC interactions, we calculated the imaginary coherence between the two areas upon exposure to either saline or octanal. While the coherence increase in RR frequency band was higher for odor-triggered events as compared to baseline events, it was similar for saline and octanal (χ^2^(2) = 23.22, *p* = 9.06 × 10^−6^, Kruskal-Wallis test, Wilcoxon rank-sum test with Bonferroni correction as post hoc test, removed outliers: baseline 1, saline 1, octanal 2) (Fig 8D and Table 1). In contrast, the coherence in fast frequencies significantly augmented in the presence of octanal when compared to saline-evoked or baseline events (theta: χ^2^(2) = 43.99, *p* = 2.81 × 10^−10^, Kruskal-Wallis test, Wilcoxon rank-sum test with Bonferroni correction as post hoc test, beta: χ^2^(2) = 48.48, *p* = 2.98 × 10^−11^, Kruskal-Wallis test, Wilcoxon rank-sum test with Bonferroni correction as post hoc test) (Fig 8D and Table 1). These data suggest that discontinuous bursts, either spontaneous or odor-induced, facilitate the long-range OB–LEC coupling and boost local entrainment in beta band of entorhinal circuits.

**Table 1:** Quantification of odor responses in neonatal OB–LEC networks (related to Fig 8).

	OB amplitude (relative change)				LEC amplitude (relative change)				OB–LEC coherence (relative change)			
	No odor	Saline	Octanal	*p*	No odor	Saline	Octanal	*p*	No odor	Saline	Octanal	*p*
RR	1.34 1.00–1.61	2.78 1.17–3.20	3.41 2.41–4.50	<0.001	1.59 1.17–3.31	2.27 1.29–4.45	5.38 2.21–8.94	0.001	0.69 0.50–1.0	1.18 0.76–1.49	1.38 0.90–1.73	<0.001
Theta	1.90 1.05–2.93	4.22 2.72–6.80	5.79 3.82–14.57	<0.001	2.01 1.48–4.43	1.19 1.48–2.71	4.87 2.64–7.18	<0.001	0.71 0.55–0.79	0.93 0.78–1.05	1.33 1.03–1.51	<0.001
Beta	1.37 1.14–1.68	2.10 1.62–3.31	5.61 4.50–9.79	<0.001	1.52 1.15–2.66	1.76 1.18–2.34	3.74 2.60–6.03	<0.001	0.61 0.42–0.76	1.03 0.91–1.19	1.32 1.19–1.44	<0.001

The values are given as median and interquartile ranges and *p*-values correspond to the difference between the three conditions (Kruskal-Wallis test).

Abbreviations: LEC, lateral entorhinal cortex; OB, olfactory bulb; RR, respiration-related rhythm.

### Chronic manipulation of olfactory periphery disrupts the development of functional coupling between OB and LEC

**Fig 9:**
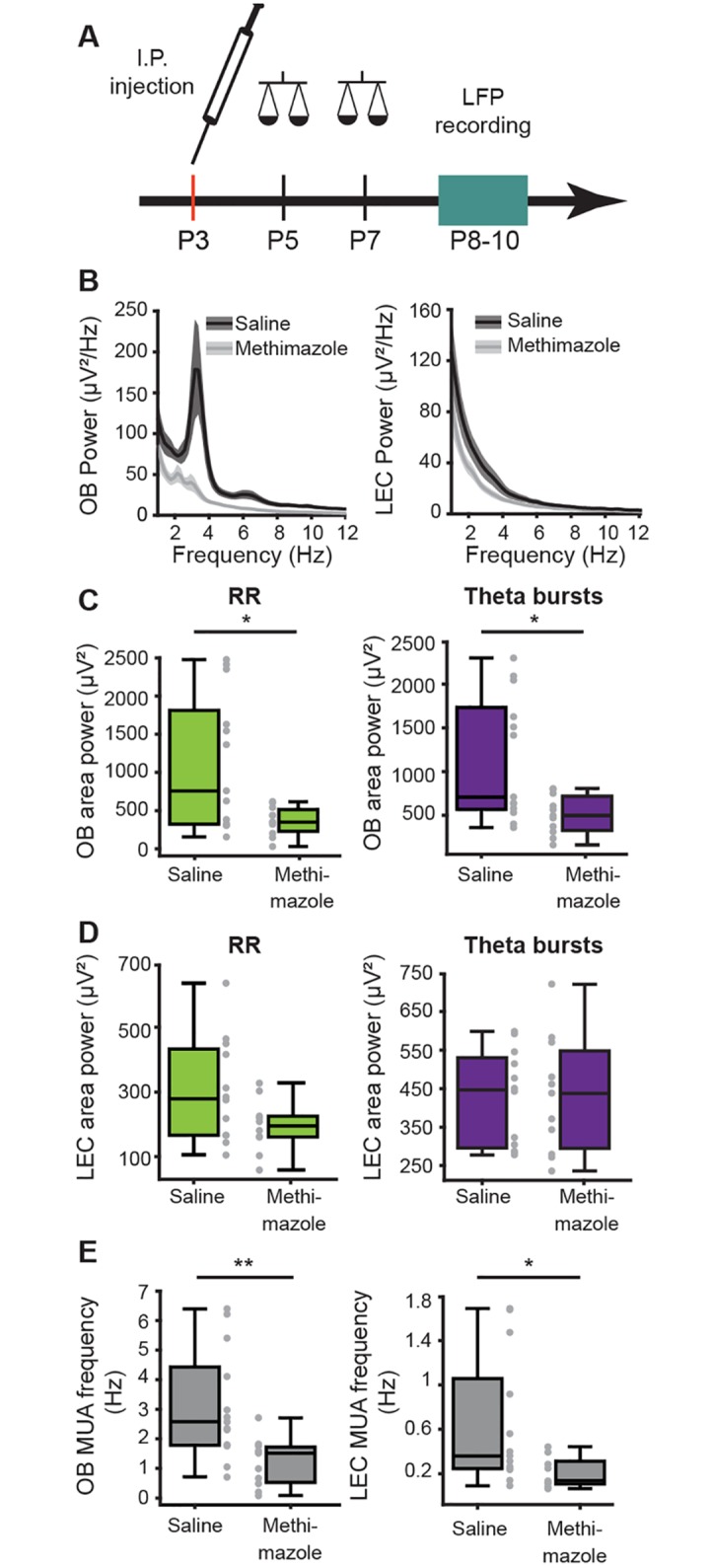
Effects of pharmacological lesioning of the nasal epithelium on the development of OB–LEC activity. (A) Schematic drawing of experimental protocol. (B) Power spectra (mean ± SEM) of LFP recorded in OB (left) and LEC (right) of methimazole- (gray) and saline-treated (black) mice. (C) Box plots displaying power of RR (green) and theta bursts (purple) recorded in OB of methimazole- (gray) and saline-treated (black) mice. (D) Same for RR and theta bursts recorded in LEC. (E) Box plots displaying MUA frequency in OB and LEC of methimazole- (gray) and saline-treated (black) animals. (Wilcoxon rank-sum test, *p < 0.05; **p < 0.01). Data are available in S1 Data. I.P., intraperitoneal; LEC, lateral entorhinal cortex; LFP, local field potential; MUA, multiunit activity; OB, olfactory bulb; RR, respiration-related rhythm.

The tight functional coupling between OB and LEC, as well as the impact of olfactory stimuli on the entorhinal activity, suggests that early activation of the olfactory periphery might be critical for the maturation of LEC as the gatekeeper of limbic circuits. To test this hypothesis, we chronically lesioned the nasal epithelium at P3 using methimazole (*n* = 11 pups) [55]. When compared to controls (i.e., saline-treated age-matched pups, *n* = 13), the power of oscillatory activity in the OB of methimazole-treated P8–10 animals was reduced for both RR (saline: median: 756.86 μV2, iqr 322.8–1,814.3; methimazole: median: 349.92 μV2, iqr 230.49–513.90, *p* = 0.0489, Wilcoxon rank-sum test) and theta bursts (saline: median: 708.38 μV2, iqr 569.1–1,731.9; methimazole: median: 497.9195 μV2, iqr 329.81–715.83, *p* = 0.0277, Wilcoxon rank-sum test) (Fig 9B and 9C). Even if methimazole decreased the oscillatory power in LEC of some pups, this effect did not reach significance level (Fig 9D). However, the neuronal firing was affected in both areas after degeneration of olfactory epithelium. The spiking frequency in MCL was lower in methimazole-treated pups when compared with saline-treated pups (saline: median: 0.52 Hz, iqr 1.79–2.58; methimazole: median: 1.52 Hz, iqr 0.53–1.72, *p* = 0.0038, Wilcoxon rank-sum test). Correspondingly, entorhinal neurons fired less after methimazole treatment (saline: median: 0.36, iqr 0.25–1.06; methimazole: median: 0.14, iqr 0.11–0.31, *p* = 0.0326, Wilcoxon rank-sum test, 2 outliers removed) (Fig 9E). These findings suggest that an intact olfactory periphery and the corresponding stimuli during the first postnatal week are necessary for the development of neuronal patterns in LEC.

## Discussion

**Fig 10:**
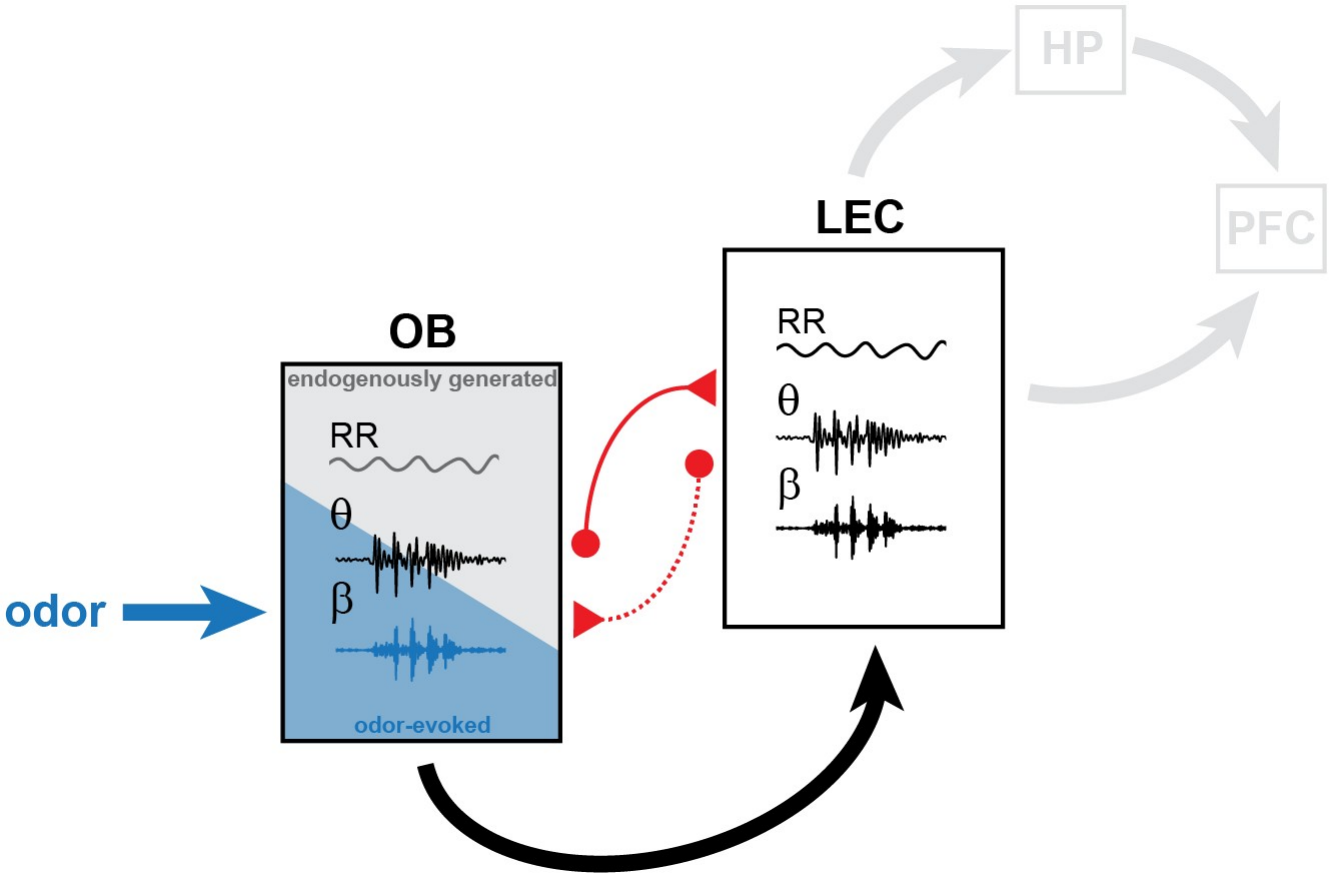
Schematic diagram of structural and functional coupling within OB–LEC networks of neonatal mice. Mutual axonal projections (red) connect neonatal OB and LEC. Dotted line corresponds to weak anatomical connectivity. In OB of neonatal mice, continuous air flow–dependent RR and discontinuous MTC-driven theta bursts represent the two major patterns of oscillatory activity. They are augmented by olfactory stimuli (blue) that additionally evoke beta oscillations. OB activity boosts the oscillatory entrainment of neonatal LEC that, in turn, might drive the limbic circuits during development. HP, hippocampus; LEC, lateral entorhinal cortex; MTC, mitral and tufted cell; OB, olfactory bulb; PFC, prefrontal cortex; RR, respiration-related rhythm.

The assembly of neurons into functional networks during development is the prerequisite for behavioral performance in adults. Entrainment of neurons into coordinated oscillatory rhythms represents a powerful assembling principle that has been initially identified to control the topographic organization of sensory systems [6,8,56,57]. More recently, patterns of coordinated activity have been characterized in the developing limbic system [9,14,15,58,59]. However, it is still unclear whether sensory and limbic circuits adhere to similar assembling principles and how they interact during early development. In the present study, we tested the hypothesis that coordinated activity patterns in the neonatal OB contribute to the oscillatory entrainment of LEC, the gatekeeper of limbic circuits during development. Combining anatomical tracing with in vivo electrophysiology, optogenetics, pharmacology, and sensory manipulations, we demonstrate that (1) two major patterns of coordinated activity entrain the neonatal OB: continuous slow frequency oscillations temporally related to respiration and discontinuous theta band oscillations critically depending on MTC activity; (2) both rhythms temporally couple the neonatal OB and LEC, with OB theta bursts boosting the oscillatory entrainment and firing within entorhinal circuits via mono- and polysynaptic projections; (3) olfactory stimuli augment oscillatory power, induce activity in fast frequency bands, and strengthen the coupling within OB–LEC circuits; and (4) olfactory activation during the first postnatal week is critical for the functional development of entorhinal circuits. These data reveal that endogenously generated and stimulus-driven activities in OB control the oscillatory entrainment of LEC (Fig 10).

Brain development has been extensively investigated in rodents because they enable insights into a time window that remains inaccessible in humans. As altricial species, rodents are born at an immature stage of brain development. They are blind and deaf, do not whisker, and have limited motor abilities during the first postnatal days. Before the onset of the ability to actively respond to sensory stimuli, coordinated activity patterns, typically characterized by rhythmic burst discharge separated by periods of quiescence, emerge endogenously. Such patterns have been described in developing somatosensory, visual, and auditory systems. Their onset, properties, and underlying mechanisms are relatively well understood. For example, retinal waves emerge before the onset of light sensitivity and vision as local patterns of coordinated activity mediated by gap junctions and cholinergic and glutamatergic circuits [60,61]. Retinal waves synaptically propagate along the visual tract to primary visual cortex [35,62,63] and are mandatory for the refinement of visual maps [64]. Similarly, cochlear burst activity emerges before the onset of hearing as a result of coordinated firing and propagates along auditory pathways [65,66]. These cochlear bursts are crucial for the establishment of precise tonotopic maps [5,56]. The precision of whisker maps in the primary somatosensory cortex seems to be equally controlled by coordinated activity evolving during postnatal development [8,67]. In the absence of a sensory periphery with bursting activity before the onset of active whisking, passive activation of whiskers is replayed within thalamocortical circuits and contributes to refinement of topographic maps [6].

At the same postnatal age, the sense of smell is of particular relevance for pup survival. At birth, the olfactory system is considered to be more mature than the other sensory systems, yet its functional development still continues postnatally. This early maturity poses the question of whether the mechanisms of organization differ between developing olfactory pathways and other sensory systems. Whereas patterns of oscillatory activity are ubiquitous in the immature brain, their features and underlying mechanisms in the olfactory system seem to be unique. On the one hand, the continuous RR is timed by respiration/air flow and largely independent of neuronal firing in OB. In contrast to other patterns of slow oscillations described in the adult brain, the RR is independent of brain state and does not change in the presence of anesthesia. On the other hand, discontinuous theta bursts result from the activation of MTCs within OB local circuits. These findings support previous observations in vitro [68]. In adult mice, network activity in theta band emerges from respiration-coupled sensory input in the glomerular layer [69], and MTCs are mainly involved in the generation of fast oscillatory activity in gamma band [70–73]. Similarly, fast rhythms are absent in the developing OB [74], and beta band activity was induced only in the presence of odors. The protracted emergence of fast oscillations might result from late integration of OB interneurons into local circuits and from age-dependent intrinsic biophysical properties of MTCs. As a consequence, it has been postulated that the developing OB encodes only first-order (e.g., odor identity) but not second-order sensory information (e.g., odor context) [75,76].

The presence of both stimulus-related and endogenous network activity raises the question of whether and, if so, how both activity types either concurrently or independently shape the maturation of the olfactory system. Already the role of spontaneous activity endogenously generated in the sensory periphery has been the subject of debate. Discontinuous OB bursts in neonatal mice might have a permissive role in the establishment of precise connectivity that is inherent in an olfactory map [77]. However, it remains unclear how the spontaneous and stimulus-evoked activities in OB create a coherent sensory representation lacking mutual perturbations. In other sensory systems, the two types of activity are temporally separated. For example, spontaneous retinal waves and cochlear bursts diminish and disappear with the onset of light sensitivity and hearing. Therefore, they do not interfere with stimulus-evoked activity. In OB, the two spontaneous and stimulus-evoked activities coexist. Elucidation of their mechanisms, as initiated in the present study, will enable us to disentangle their function(s) along the developing olfactory pathway.

In sharp contrast to most sensory pathways, the olfactory system bypasses a thalamic relay and directly conveys information from OB to cortical areas. Much research has focused on the PIR, where the bulbar topography is largely discarded and dense inputs from OB are integrated to form odor percepts [78–81]. However, also LEC neurons respond to odors [82,83] and have been proposed to act as a modulator of olfactory coding through interactions with the PIR [24,84,85]. The present results show that already at neonatal age, a tight coupling links the OB with LEC. MC axons target layer I of neonatal LEC as previously shown for adults [86,87]. These projections mediate the coupling by synchrony between the two areas as well as the early drive from OB to LEC. The neonatal LEC and OB show similar patterns of oscillatory activity, RR, and theta bursts, albeit with lower power in LEC. The coupling by synchrony between the two areas peaked within the same frequency bands, 2–4 Hz and 4–12 Hz. Given the measures used for the assessment of synchrony, it is unlikely that similarities result from volume conduction. Reflecting the more pronounced OB-to-LEC innervation as compared with feedback projections, the entorhinal firing was more strongly timed by the phase of RR and theta bursts in the OB than the OB firing was driven by the entorhinal activity. The cross-covariance analysis of spike trains demonstrated that the OB drives LEC via monosynaptic and polysynaptic projections. Therefore, OB controls LEC not only directly but also via additional brain areas such as PIR, which is driven by OB and strongly coupled with LEC. Medial septum might be another brain area involved in the indirect OB–LEC coupling, since it has been shown to control the synchronization of sniffing frequency and limbic theta oscillations [88]. This multifold gating may augment the efficiency of olfactory control on limbic circuits. In the long term, the olfactory control seems to shape these circuits during development. Chronic lesioning of olfactory receptors during the first postnatal week that decreases the ability of neonatal pups to process olfactory information diminished the entorhinal firing. Therefore, even though MC axonal projections reach the entorhinal cortex prenatally, the functional coupling within OB–LEC networks is still refined during the first postnatal week.

Although feedback projections from LEC (and PIR) to OB emerge early in life, they do not play a role in the entrainment of neonatal activity, as monosynaptic top-down coupling was absent. Possibly, this is due to the delayed development of the OB interneuronal circuitry, which receives most of the top-down projections. Hence, their function seems to mature postnatally to reach the anticipatory top-down modulation and optimal input discrimination that have been identified at adult stage [33]. Recent findings revealed that the cellular substrate of feedforward and feedback interactions between OB, LEC, and PIR of adult mice are highly complex [24]. We hypothesize that, under the influence of excitatory inputs from OB, the local entorhinal circuitry is activated. MC axons target layer I, where they terminate on dendrites of multipolar and pyramidal cells based in layers II and III [20], suggesting that coordinated OB activity may cause an overall excitation in LEC that might facilitate the formation and refinement of local circuits.

Olfactory information reaches the adult HP (CA1 and dentate gyrus) via reelin-positive neurons in LEC [24,89]. Along these axonal projections, the oscillatory activity is synchronized and enables directed functional interactions between OB, LEC, and HP. In turn, HP unidirectionally projects to PFC. At a functional level, the communication across areas involves oscillatory activity that temporally coordinates the neuronal assemblies. For example, respiration-related slow activity has recently been found to occur simultaneously with theta oscillations [90] and moreover entrain faster beta and gamma oscillations in LEC, HP, and PFC [45,91,92]. Taking into account the role of HP and PFC for cognitive processing [93], the OB activity that directly entrains the limbic circuit via LEC activation might represent a powerful control mechanism of memory and executive performance of adult [94,95].

It is tempting to speculate about the potential functions of OB-driven entrainment of LEC during neonatal development, before the emergence of cognitive abilities. Our previous results demonstrated that LEC acts as gatekeeper of prefrontal–hippocampal interactions shortly after birth [11]. Discontinuous theta bursts in LEC drive the oscillatory entrainment and time the firing of both prelimbic subdivision of PFC and CA1 area of the intermediate/ventral HP (Fig 10). Here, we show that MTC-dependent theta activity of neonatal OB boosts RR and theta bursts in LEC. On the other hand, olfactory stimuli elicit even faster entrainment of OB–LEC circuitry, with beta band oscillations being only detectable in the presence of odors, such as octanal. This is in line with LEC–hippocampal coupling at beta frequency in adult rats during odor learning [96]. An important issue that remains to be elucidated is whether specific scents that the pups naturally encounter during development, such as maternal odors, shape the network function even stronger than “artificial” odors. The effects of maternal odor on physical, neuroendocrine, and behavioral development of pups have been extensively investigated [27,97,98], yet very little is known about the underlying cellular and circuit mechanisms. We propose that endogenously generated and odor-evoked OB activity, especially as a result of maternal odor, might increase the level of excitability within entorhinal–prelimbic–hippocampal networks and strengthen their wiring. By these means, the olfactory system could facilitate the postnatal maturation of limbic circuitry and, ultimately, the emergence of cognitive abilities.

## Materials and methods

### Ethics statement

All experiments were performed in compliance with the German laws (Tierschutzgesetz) and the guidelines of the European Union for the use of animals in research (European Union Directive 2010/63/EU) and were approved by the local ethical committee (Behörde für Gesundheit und Verbraucherschutz, ID 15/17).

### Experimental model and subject details

#### Mice

Timed-pregnant C57Bl/6J and Tbet-cre mice from the animal facility of the University Medical Center Hamburg-Eppendorf as well as B6.Cg-*Gt(ROSA)26Sor*^*tm40*.*1(CAG-aop3/EGFP)Hze*^/J mice (Ai40[RCL-ArchT-EGFP]-D, Jackson Laboratory, stock no.: 02118) and Tbet-cre;ArchT-EGFP mice (bred by the animal facility of the University Medical Center Hamburg-Eppendorf) were housed individually in breeding cages at a 12 h light / 12 h dark cycle and fed ad libitum. Mouse lines used for CLARITY experiments (Tbet-cre mice, B6.Cg-*Gt(ROSA)26Sor*^*tm9(CAG-tdTomato)Hze*^/J (Ai9(RCL-tdT), Jackson Laboratory, stock no: 007909 and Tbet-cre;tdT mice) were bred in the animal facility at RWTH Aachen University under similar conditions. The day of vaginal plug detection was defined as E0.5, and the day of birth was assigned as P0. Male mice underwent sensory manipulation, light stimulation, pharmacological treatment, and multisite electrophysiological recordings at P8–10. For CLARITY experiments, male and female mice were used. Genotypes were determined using genomic DNA and following primer sequence (Metabion, Planegg/Steinkirchen, Germany): for Cre in Ai40(RCL-ArchT-EGFP)-D mice: PCR forward primer 5′-ATCCGAAAAGAAAACGTTGA-3′ and reverse primer 5′-ATCCAGGTTACGGATATAGT-3′; for ROSA26-wt PCR forward primer 5′-AAAGTCGCTCTGAGTTGTTAT-3′ and reverse primer 5′-GGAGCGGGAGAAATGGATATG-3′; for GFP-tg PCR forward primer 5′-CTGGTCGAGCTGGACGGCGACG-3′ and reverse primer 5′-GTAGGTCAGGGTGGTCACGAG-3′; for Cre in Ai9(RCL-tdT) mice: forward primer 5′-CATGTCCATCAGGTTCTTGC-3′ and reverse primer 5′-AGAGAAAGCCCAGGAGCAG-3′; for tdTomato forward primer 5′-GGCATTAAAGCAGCGTATCC-3′ and reverse primer 5′ CTGTTCCTGTACGGCATGG-3′. The PCR reactions were as follows: 10 min at 95 °C, 30 cycles of 45 s at 95 °C, 90 s at 54 °C, and 90 s at 72 °C, followed by a final extension step of 10 min at 72 °C (Cre-tg and ROSA26-wt), 10 min at 95 °C, 30 cycles of 45 s at 95 °C, 90 s at 68 °C, and 90 s at 72 °C, followed by a final extension step of 10 min at 72 °C (GFP-tg). In addition to genotyping, EGFP expression in OB prior to surgery was detected using a dual fluorescent protein flashlight (Electron microscopy sciences, Hatfield, PA, USA).

### Surgical procedures

#### Surgical preparation for electrophysiology and light delivery in vitro

For patch-clamp recordings, pups were decapitated, and brains were sliced in 300 μm–thick coronal sections. Slices were incubated in oxygenated ACSF containing (in mM) 119 NaCl, 2.5 KCl, 1 NaH_2_PO_4_, 26.2 NaHCO_3_, 11 glucose, 1.3 MgSO_4_ (320 mOsm) at 37 °C. Prior to recordings, slices were maintained at room temperature and superfused with oxygenated ACSF.

#### Surgical preparation for electrophysiology and light delivery in vivo

For recordings in nonanesthetized state, 0.5% bupivacain / 1% lidocaine was locally applied on the neck muscles. For recordings under anesthesia, mice were injected i.p. with urethane (1 mg/g body weight; Sigma-Aldrich, St. Louis, MO, USA) prior to surgery. For both groups, under isoflurane anesthesia (induction: 5%, maintenance: 2.5%) the head of the pup was fixed into a stereotaxic apparatus as previously reported [9].

#### Viral transfection of MTCs

Transfection of MTCs with a ChR2 derivate was achieved by injecting 200–400 μl of the construct (pAAV-Ef1a-DIO hChR2(E123T/T159C)-EYFP, 100 μl at a titer ≥ 1 × 10^13^ vg/mL, Addgene, Watertown, MA, USA) unilaterally in the OB of P0–1 pups.

The surgery protocols are described in detail in the Supporting Information.

### Electrophysiology

#### Electrophysiological recordings in vivo

One-shank electrodes (NeuroNexus, MI, USA) with 16 recording sites were inserted into dorsal (depth 0.5–1.2 mm, angle 0°) or ventral OB (1.4–1.8 mm, angle 0°) as well as in LEC (depth: 2 mm, angle: 10° from the vertical plane). Two-shank optoelectrodes (Buzsaki16-OA16LP, NeuroNexus, Ann Arbor, MI, USA) with 8 recordings sites on each shank aligned with an optical fiber ending 40 μm above the top recording site were inserted into ventral OB. Extracellular signals were band-pass filtered (0.1 Hz–9 kHz) and digitized (32 kHz) by a multichannel amplifier (Digital Lynx SX; Neuralynx, Bozeman, MT, USA) and Cheetah acquisition software (Neuralynx).

#### Electrophysiological recordings in vitro

Whole-cell patch-clamp recordings were performed from MCs identified by their location in the MCL and visualized by membrane-bound EGFP. All recordings were performed at room temperature. Recording electrodes (4–9 MΩ) were filled with K-gluconate based solution containing (in mM): 130 K-gluconate, 10 HEPES, 0.5 EGTA, 4 Mg-ATP, 0.3 Na-GTP, 8 NaCl (285 mOsm [pH 7.4]), and 0.5% biocytin for post hoc morphological identification of recorded cells. Recordings were controlled with the Ephus software [99] in the MATLAB environment (The MathWorks, Natick, MA, USA).

### Morphological investigation

#### CLARITY

Brains from neonatal mice of both sexes were sliced in 1 mm–(for LEC) and 500 μm–thick (for OB) coronal sections. To maintain the structural integrity, the tissue was fixed overnight at 4 °C in hydrogel fixation solution containing 4% acrylamide, 0.05% bis-acrylamide, 0.25% VA-044 Initiator, 4% PFA in PBS−/−. After polymerization and embedding, the nuclear marker DRAQ5 (1:1,000) was added to the samples. After washing steps, the samples were incubated for 24 h in RIMS80 containing 80 g Nycodenz, 20 mM PS, 0.1% Tween 20, and 0.01% sodium acid.

#### Retrograde tracing

For retrograde tracing, anesthetized P3–4 mice received unilateral FG (Fluorochrome, Denver, CO, USA) injections into OB (0.8 mm anterior from the frontonasal suture, 0.8 mm from midline) or LEC (0 mm posterior to bregma, 5 mm from midline). After 4–5 d, pups were deeply anesthetized and perfused at P8.

All staining protocols are described in detail in the Supporting Information.

### Manipulations

#### Light stimulation in vitro

Whole-cell current-clamp recordings were performed from ArchT-EGFP- or Chr2-EYFP-expressing MTCs in coronal slices from neonatal Tbet-cre;ArchT mice or Tbet-cre mice transfected with a cre-dependent virus carrying ChR2. Yellow light pulses (595 nm) of different light intensities (1.5–19.3 mW mm^−2^) were applied to test the effect of ArchT stimulation on the membrane potential. Trains of blue light pulses (470 nm, 3 ms) of different frequencies where applied to induce action potential firing in ChR2-transfected MTCs.

#### Light stimulation in vivo

For light-induced inhibition of MTC activity, trapezoid light stimulation was applied using a diode-pumped solid-state (DPSS) laser (Cobolt Mambo, 594 nm, Omicron Laserage, Rodgau-Dudenhofen, Germany). For activation of MTCs, pulsed (laser on-off) light stimulations generated with a diode laser (473 nm; Omicron Laserage, Rodgau-Dudenhofen, Germany) were used. Resulting light power was in the range of 38.2–103.8 mW mm^−2^ at the tip of optical fiber. Both lasers were controlled with an arduino uno (Arduino, Ivrea, Italy).

#### Naris occlusion

One naris was closed using silicon adhesive (Kwik-Sil, World Precision Instruments, Sarasota, FL, USA). After a recovery period of 5 min, the recording was pursued while one naris was sealed.

#### Pharmacological inactivation

To block the firing of OB neurons, lidocaine hydrochloride 4% in 0.9% NaCl, ([pH 7.0] with NaOH) was slowly infused into the OB.

#### Odor stimulation

An eight-channel dilution olfactometer (Aurora Scientific, Aurora, ON, Canada) was used for stimulus delivery.

#### Lesion of nasal epithelium

Methimazole (Sigma-Aldrich, 100 mg/kg in sterile saline) or saline was injected i.p. at P3.

All manipulation protocols are described in detail in the Supporting Information.

### Quantification and statistical analysis

#### Immunohistochemistry quantification

Images were analyzed using ImageJ.

#### Detection of respiration frequency

Respiration was monitored using a piezo-electric sensor placed under the pup’s chest.

#### LFP analysis

Data were analyzed offline using custom-written scripts in the MATLAB environment (Version 9, MathWorks, Natick, MA, USA).

For details, see the Supporting Information.

#### Statistics

Statistical analysis was performed using SPSS Statistics 22 (IBM, Armonk, NY, USA) or MATLAB. Gaussian distribution of the data was assessed using the Kolmogorov-Smirnov test. None of the data sets were normally distributed. Therefore, data were tested for significance using Wilcoxon signed-rank test (2 related samples), Wilcoxon rank-sum test (2 unrelated samples), Friedman test (>2 related samples; Wilcoxon signed-rank post hoc test with Bonferroni correction), and Kruskal-Wallis test (>2 unrelated samples; Wilcoxon rank-sum test post hoc test with Bonferroni correction). Differences in proportions were tested using χ^2^ test. For classification of single-unit responses to light stimulation, significant firing rate changes were assessed statistically using Wilcoxon signed-rank test. Data are represented as median and iqr. Values were considered as outliers and removed when their distance from the 25th or 75th percentile exceeded 1.5 times the interquartile interval.

## Supporting information

S1 Fig (Related to Fig 1). **Top-down connectivity between OB and LEC in neonatal mice**. (A) Photographs of a 50 μm–thick coronal section from a P8 mouse depicting retrogradely labeled neurons in LEC (middle) after injection of FG into OB (100 μm–thick coronal section, left) at P4. Right, counterstaining for CamKII of the same section. (B) Coronal section shown in (A) when displayed at higher magnification. CamKII staining (left) enables identification of LEC sublayers IIa and IIb as well as laminar dissecans (“l.d.”). FG-labeled cell bodies (middle) have been found in layers IIa and IIb as well as layer III. Most but not all FG-labeled cells overlapped with CamKIII staining (right). FG, Fluorogold; LEC, lateral entorhinal cortex; OB, olfactory bulb; P, postnatal day; PIR, piriform cortex; rf, rhinal fissure.
(TIF)

S2 Fig (Related to Fig 2). **Reversal of RR and theta burst activity in OB of neonatal mice**. (A) Digital photomontage reconstructing the track of the DiI-labeled recording electrode in the ventral OB of a P10 mouse in a 100 μm–thick coronal section stained with green fluorescent Nissl. (B) Laminar recording of band-pass (2–4 Hz) LFP activity in OB accompanied by respiration as detected by a piezo-electric sensor. Gray boxes indicate the exhalation period. (C) Left, laminar recording of band-pass (4–12 Hz) LFP activity in OB. Right, LFP activity (gray dotted box) shown at larger magnification. Red line marks signal reversal. EPL, external plexiform layer; GCL, granule cell layer; GL, glomerular layer; IPL, internal plexiform layer; LFP, local field potential; MCL, mitral cell layer; OB, olfactory bulb; P, postnatal day.
(TIF)

S3 Fig (Related to Fig 2). **Characterization of activity patterns in the dorsal and ventral OB of neonatal mice**. (A) Left, digital photomontage reconstructing the track of the DiI-labeled recording electrode (red) in the dorsal OB of a green fluorescent Nissl-stained 100 μm–thick coronal section. The gray dots correspond to multiple recording sites spanning all OB layers. Right, the corresponding LFP recording of the oscillatory activity in EPL of a P10 mouse displayed band-pass filtered and accompanied by the wavelet spectrogram. (B) Same as A for ventral OB. (C) Box plots displaying the power of RR (green) and theta bursts (purple) in the dorsal and ventral OB. (D) Box plots displaying the occurrence, duration, and amplitude of theta bursts in dorsal and ventral OB. (E) Box plots displaying the mean imaginary coherence of RR (green) and theta bursts (purple) between dorsal OB and LEC as well as between ventral OB and LEC. In (C)–(E), gray dots correspond to individual animals. (Wilcoxon rank-sum test). Data are available in S1 Data. EPL, external plexiform layer; GCL, granule cell layer; GL, glomerular layer; IPL, internal plexiform layer; LFP, local field potential; MCL, mitral cell layer; OB, olfactory bulb; P, postnatal day.
(TIF)

S4 Fig (Related to Fig 2). **Effects of urethane anesthesia on the network activity in neonatal OB**. (A) Power spectra (mean ± SEM) of LFP recorded in the neonatal OB before (blue) and during (red) urethane anesthesia when calculated for the entire trace (left) and for concatenated time windows of theta bursts (right). Insets, box plots displaying RR and theta area power before and during urethane anesthesia (*n* = 12, 1 outlier removed). (B) Box plots displaying the occurrence and duration of theta bursts as well as the level of discontinuity of theta bursts measured as fraction of recording time with activity in theta band in neonatal OB (*n* = 18). Gray dots and lines correspond to individual animals. (*p < 0.05; ***p < 0.001, Wilcoxon signed-rank test). Data are available in S1 Data. LFP, local field potential; OB, olfactory bulb; P, postnatal day; RR, respiration-related rhythm.
(TIF)

S5 Fig (Related to Fig 3). **Optogenetic silencing of MCs in vitro**. (A) Top, digital photomontage reconstructing by high-resolution confocal imaging the morphology of a biocytin-filled (red), EGFP-stained (green) ArchT-positive MC in the OB of a P9 mouse. Bottom, same MC displayed at higher magnification. (B) Whole-cell current-clamp recording of an MC in the OB of a P10 mouse before (top) and during repeated stimulation with 3 s–long yellow light pulses (bottom). Note that the light caused hyperpolarization of RMP and abolished firing. (C) Whole-cell current-clamp recordings of an MC in the OB of a P10 mouse during simultaneous light stimulation (595 nm, 3 s yellow) and current injection (60 pA, 3 s) meant to mimic synaptic inputs. Note the efficient silencing of firing even in the presence of a depolarizing current pulse. EGFP, enhanced green fluorescent protein; MC, mitral cell; OB, olfactory bulb; P, postnatal day; RMP, resting membrane potential.
(TIF)

S6 Fig (Related to Fig 3). **Membrane potential oscillations of MCs in vitro**. Top, representative current-clamp recording from a P9 MC (−48 mV) showing membrane potential oscillations. Bottom, power spectrum (blue, mean ± SEM) of membrane voltage oscillations of MCs with firing rates < 0.1 Hz (blue). Power spectra of individual MCs are shown in gray. MC, mitral cell.
(TIF)

S7 Fig (Related to Fig 4). **Effects of urethane anesthesia on the network activity in neonatal LEC**. (A) Power spectra (mean ± SEM) of LFP recorded in the neonatal LEC before (blue) and during (red) urethane anesthesia when calculated for the entire trace (left) and for concatenated time windows of theta bursts (right). Insets, box plots displaying RR and theta power before and during urethane anesthesia (*n* = 12, 1 outlier removed, *n* = 13). (B) Box plots displaying the occurrence and duration of theta bursts as well as the level of discontinuity of theta bursts measured as a fraction of recording time with activity in theta band in neonatal LEC (*n* = 18). Gray dots and lines correspond to individual animals. (*p < 0.05; ***p < 0.001; Wilcoxon signed-rank test). Data are available in S1 Data. LEC, lateral entorhinal cortex; LFP, local field potential; RR, respiration-related rhythm.
(TIF)

S8 Fig (Related to Fig 7). **Optogenetic activation of MCs in vitro**. (A) Top, digital photomontage reconstructing by high-resolution confocal imaging the morphology of a biocytin-filled (red), EYFP-stained (green) ChR2-positive MC in the OB of a P6 mouse. Bottom, same MC displayed at higher magnification. (B) Representative voltage responses of a transfected MC to trains of 3 ms–long light stimuli at different frequencies. (C) Bar diagram displaying the mean firing probability of transfected neurons in response to repetitive light stimulation at different frequencies (*n* = 4 neurons). Data are available in S1 Data. ChR2, channelrhodopsin 2; EYFP, enhanced yellow fluorescent protein; MC, mitral cell; OB, olfactory bulb; P, postnatal day.
(TIF)

S9 Fig (Related to Fig 7). **Light-evoked spike responses of MTC in relationship to network oscillations**. (A) Raw signal, band-pass-filtered LFP (1–100 Hz, theta 4–12 Hz), MUA (<400 Hz), and SUA spike trains before, during, and after pulsed (8 Hz) light stimulation (473 nm) of MTCs in OB of a P8 Cre+ Tbet-cre mouse. (B) Same as (A, dotted box) displayed at larger timescale. LFP, local field potential; MTC, mitral and tufted cell; MUA, multiunit activity; OB, olfactory bulb; P, postnatal day; SUA, single-unit activity.
(TIF)

S10 Fig Cross-covariance analysis as a measure of directionality of interactions within OB–LEC–PIR circuits. (A) Left, line plots showing smoothed mean standardized cross-covariance of spike pairs recorded from OB and LEC (*n* = 251), OB and PIR (*n* = 62), and LEC and PIR (*n* = 142) that have significant coupling at lags between −50 and 50 ms (black dotted lines correspond to significance threshold). Right, same plot displayed at higher magnification to highlight the cross-covariance peaks for OB–LEC and OB–PIR spike trains. (B) Schematic overview of mono- and polysynaptic connectivity between OB, LEC, and PIR as revealed by cross-covariance analysis of spike trains. (C) Pie charts showing the percentage of monosynaptically coupled, polysynaptically coupled, and uncoupled unit pairs. LEC, lateral entorhinal cortex; OB, olfactory bulb; PIR, piriform cortex.
(TIF)

S1 Table (Related to S4 and S7 Figs). **Effect of urethane anesthesia on activity patterns in neonatal OB and LEC**. The values are given as medians and interquartile ranges, and significant differences are shown as *p < 0.05, ***p < 0.001 (Wilcoxon signed-rank test). LEC, lateral entorhinal cortex; OB, olfactory bulb.
(DOCX)

S1 Data Excel spreadsheet containing, in separate sheets, the underlying data for Figs 2D, 2E, 3E, 3F, 4D, 4E, 5B, 5E, 6D, 6E, 6F, 6G, 7C, 7D, 7E, 7F, 7G, 8B, 8C, 8D, 9C, 9D and 9E, S3C, S3D, S3E, S4A, S4B, S7A, S7B and S8C Figs. Removed outliers are marked in red.
(XLSX)

S1 Text Full description of all methods, including references. (DOCX)

## Abbreviations

af: amygdaloid fissure
ChR2: channelrhodopsin 2
EGFP: enhanced green fluorescent protein
EPL: external plexiform layer
EYFP: enhanced yellow fluorescent protein
FG: Fluorogold
GCL: granule cell layer
HP: hippocampus
i.p.: intraperitoneal
IPL: internal plexiform layer
iqr: interquartile range
LEC: lateral entorhinal cortex
LFP: local field potential
LOT: lateral olfactory tract
MC: mitral cell
MCL: mitral cell layer
MTC: mitral and tufted cell
MUA: multiunit activity
OB: olfactory bulb
OMI: optogenic modulation index
P: postnatal day
PCA: principal component analysis
PFC: prefrontal cortex
PIR: piriform cortex
PPC: pairwise phase consistency
PRh: perirhinal cortex
rf: rhinal fissure
RR: respiration-related rhythm
SUA: single-unit activity
