# Supplementary figures and images for: Coordinated electrical activity in the olfactory bulb gates the oscillatory entrainment of entorhinal networks in neonatal mice
Source: PLoS Biol. 2019 Jan 31;17(1):e2006994. doi: 10.1371/journal.pbio.2006994 (PMC6354964; doi:10.1371/journal.pbio.2006994)

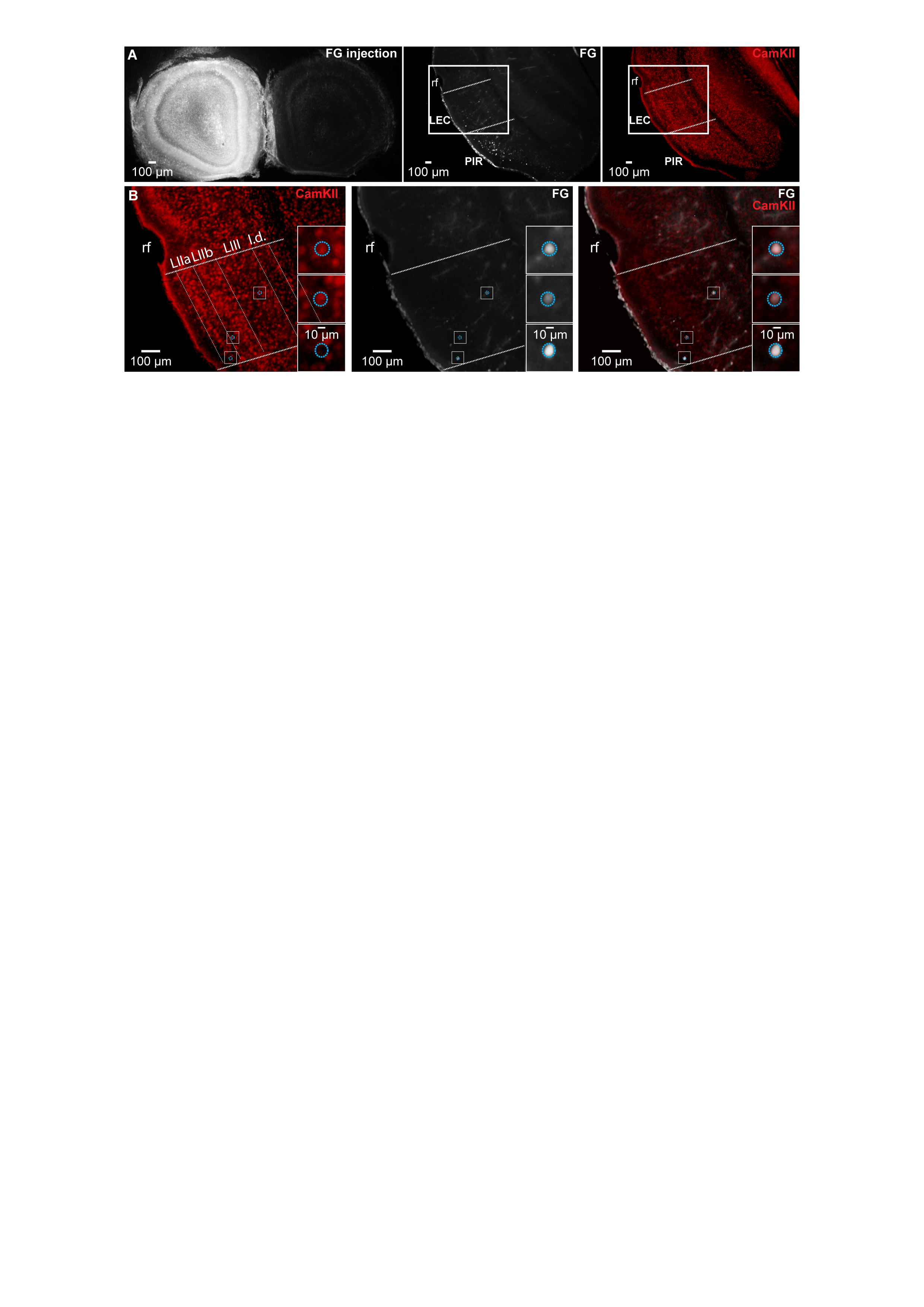

Supplement: S1 Fig — Top-down connectivity between OB and LEC in neonatal mice. (A) Photographs of a 50 μm–thick coronal section from a P8 mouse depicting retrogradely labeled neurons in LEC (middle) after injection of FG into OB (100 μm–thick coronal section, left) at P4. Right, counterstaining for CamKII of the same section. (B) Coronal section shown in (A) when displayed at higher magnification. CamKII staining (left) enables identification of LEC sublayers IIa and IIb as well as laminar dissecans (“l.d.”). FG-labeled cell bodies (middle) have been found in layers IIa and IIb as well as layer III. Most but not all FG-labeled cells overlapped with CamKIII staining (right). FG, Fluorogold; LEC, lateral entorhinal cortex; OB, olfactory bulb; P, postnatal day; PIR, piriform cortex; rf, rhinal fissure. (TIF) [file pbio.2006994.s001.tif]

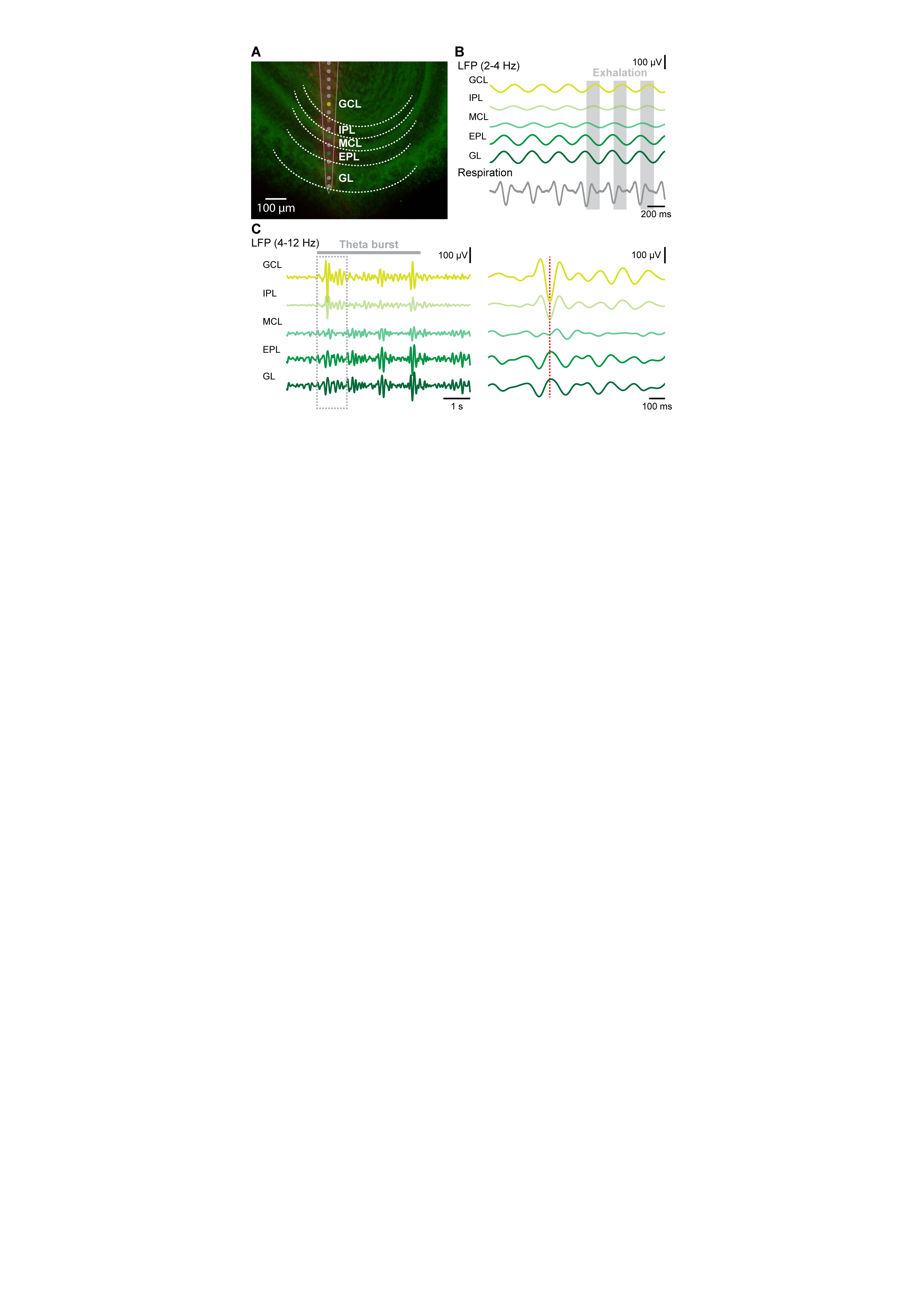

Supplement: S2 Fig — Reversal of RR and theta burst activity in OB of neonatal mice. (A) Digital photomontage reconstructing the track of the DiI-labeled recording electrode in the ventral OB of a P10 mouse in a 100 μm–thick coronal section stained with green fluorescent Nissl. (B) Laminar recording of band-pass (2–4 Hz) LFP activity in OB accompanied by respiration as detected by a piezo-electric sensor. Gray boxes indicate the exhalation period. (C) Left, laminar recording of band-pass (4–12 Hz) LFP activity in OB. Right, LFP activity (gray dotted box) shown at larger magnification. Red line marks signal reversal. EPL, external plexiform layer; GCL, granule cell layer; GL, glomerular layer; IPL, internal plexiform layer; LFP, local field potential; MCL, mitral cell layer; OB, olfactory bulb; P, postnatal day. (TIF) [file pbio.2006994.s002.tif]

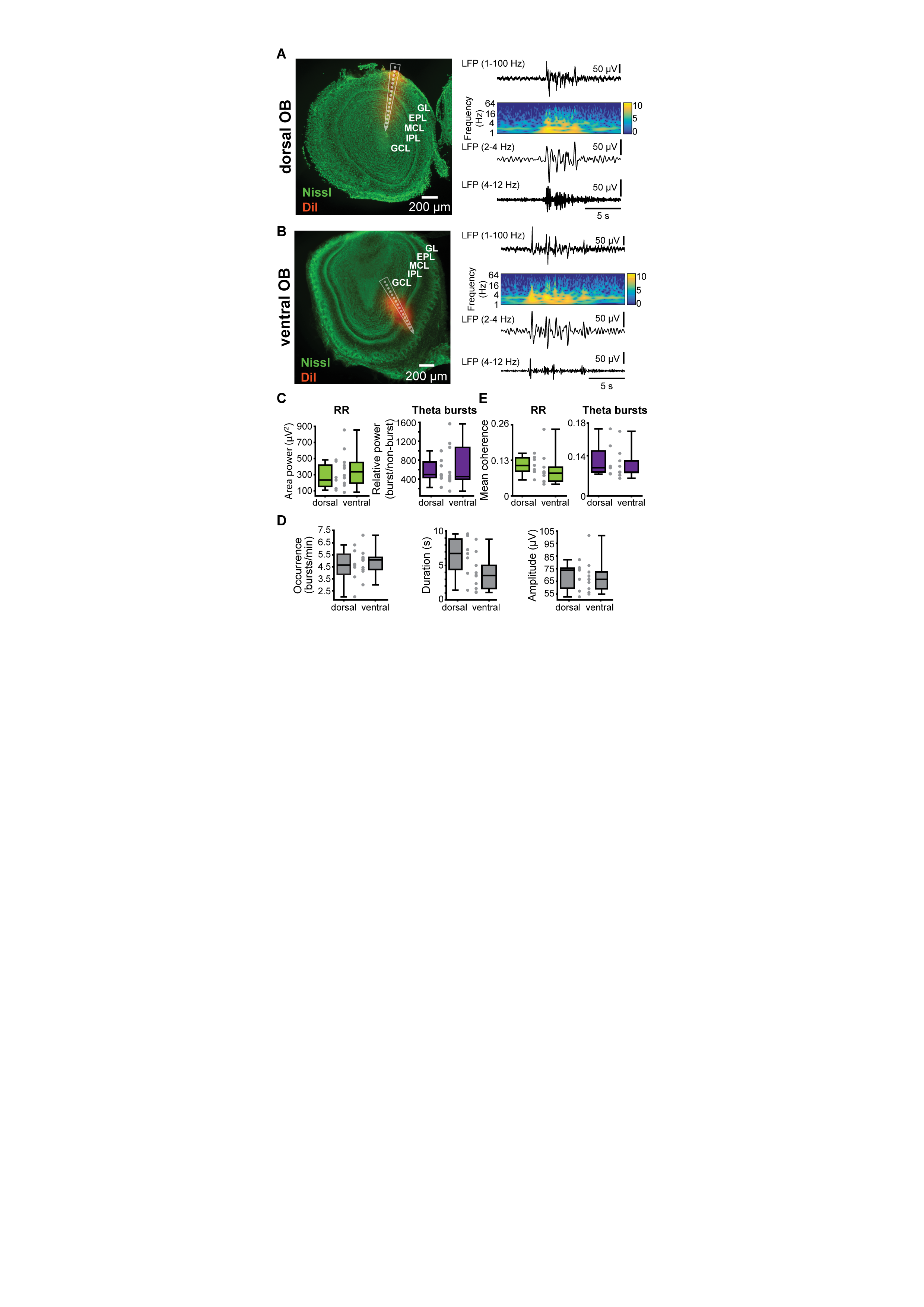

Supplement: S3 Fig — Characterization of activity patterns in the dorsal and ventral OB of neonatal mice. (A) Left, digital photomontage reconstructing the track of the DiI-labeled recording electrode (red) in the dorsal OB of a green fluorescent Nissl-stained 100 μm–thick coronal section. The gray dots correspond to multiple recording sites spanning all OB layers. Right, the corresponding LFP recording of the oscillatory activity in EPL of a P10 mouse displayed band-pass filtered and accompanied by the wavelet spectrogram. (B) Same as A for ventral OB. (C) Box plots displaying the power of RR (green) and theta bursts (purple) in the dorsal and ventral OB. (D) Box plots displaying the occurrence, duration, and amplitude of theta bursts in dorsal and ventral OB. (E) Box plots displaying the mean imaginary coherence of RR (green) and theta bursts (purple) between dorsal OB and LEC as well as between ventral OB and LEC. In (C)–(E), gray dots correspond to individual animals. (Wilcoxon rank-sum test). Data are available in S1 Data. EPL, external plexiform layer; GCL, granule cell layer; GL, glomerular layer; IPL, internal plexiform layer; LFP, local field potential; MCL, mitral cell layer; OB, olfactory bulb; P, postnatal day. (TIF) [file pbio.2006994.s003.tif]

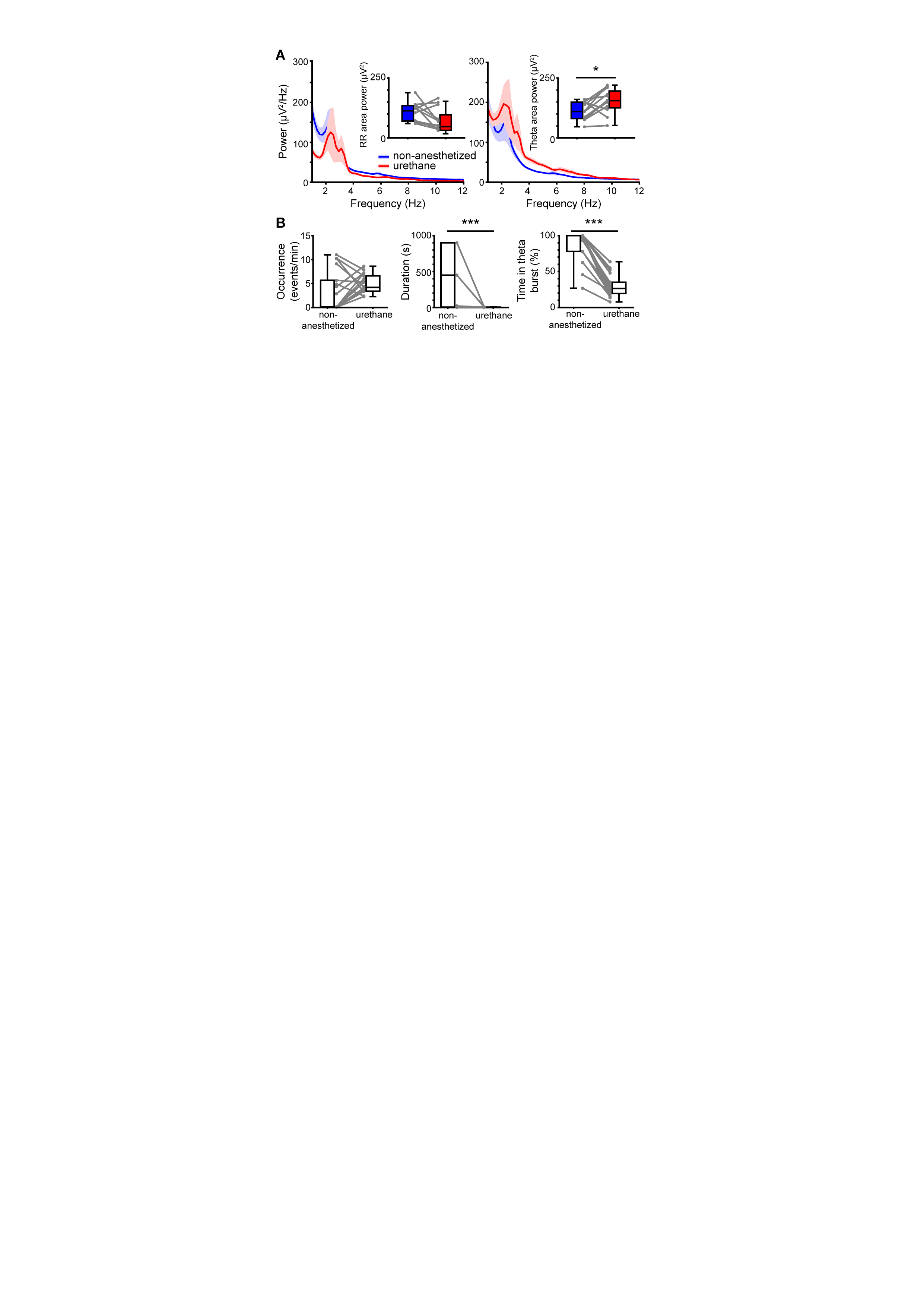

Supplement: S4 Fig — Effects of urethane anesthesia on the network activity in neonatal OB. (A) Power spectra (mean ± SEM) of LFP recorded in the neonatal OB before (blue) and during (red) urethane anesthesia when calculated for the entire trace (left) and for concatenated time windows of theta bursts (right). Insets, box plots displaying RR and theta area power before and during urethane anesthesia (n = 12, 1 outlier removed). (B) Box plots displaying the occurrence and duration of theta bursts as well as the level of discontinuity of theta bursts measured as fraction of recording time with activity in theta band in neonatal OB (n = 18). Gray dots and lines correspond to individual animals. (*p < 0.05; ***p < 0.001, Wilcoxon signed-rank test). Data are available in S1 Data. LFP, local field potential; OB, olfactory bulb; P, postnatal day; RR, respiration-related rhythm. (TIF) [file pbio.2006994.s004.tif]

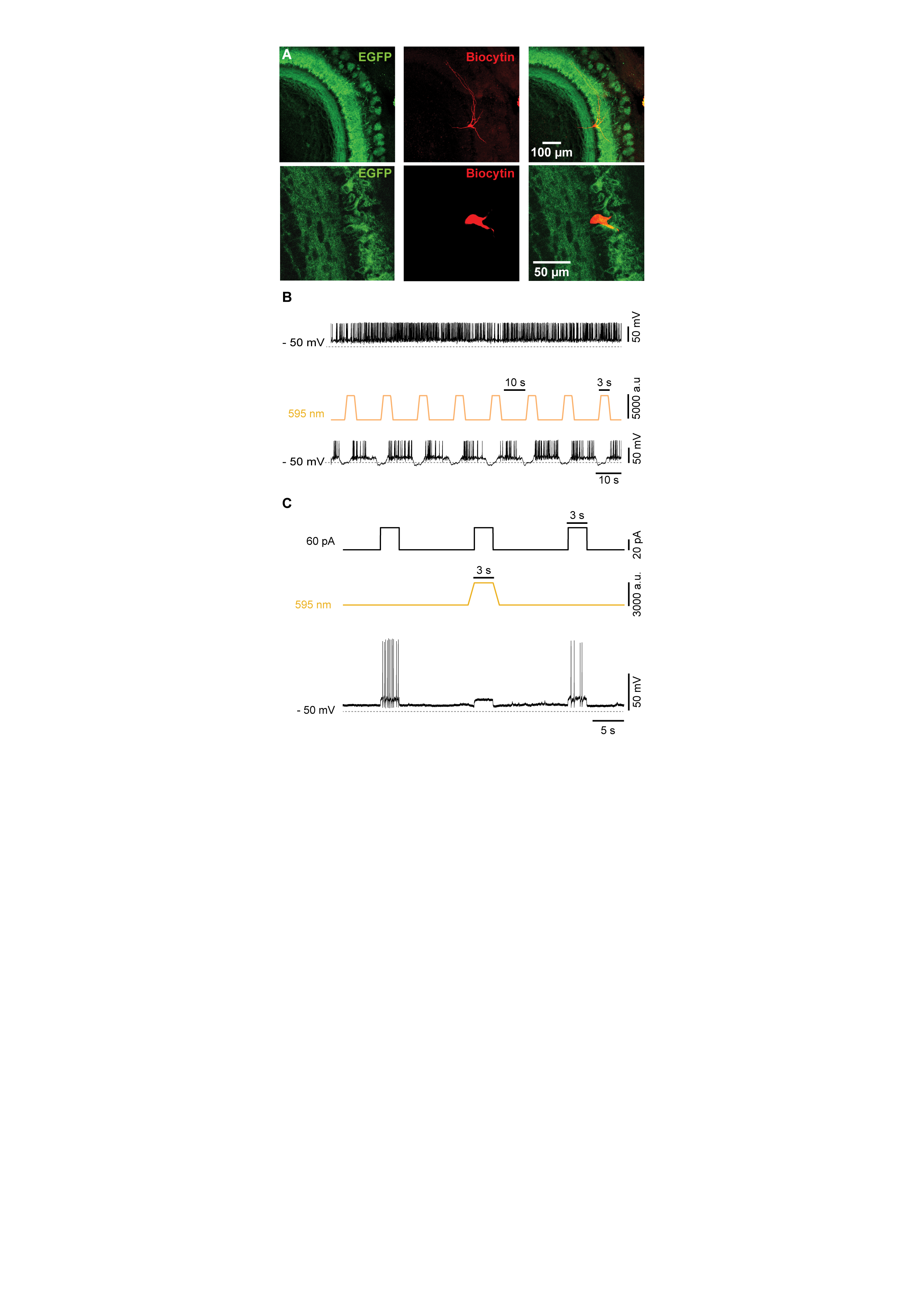

Supplement: S5 Fig — Optogenetic silencing of MCs in vitro. (A) Top, digital photomontage reconstructing by high-resolution confocal imaging the morphology of a biocytin-filled (red), EGFP-stained (green) ArchT-positive MC in the OB of a P9 mouse. Bottom, same MC displayed at higher magnification. (B) Whole-cell current-clamp recording of an MC in the OB of a P10 mouse before (top) and during repeated stimulation with 3 s–long yellow light pulses (bottom). Note that the light caused hyperpolarization of RMP and abolished firing. (C) Whole-cell current-clamp recordings of an MC in the OB of a P10 mouse during simultaneous light stimulation (595 nm, 3 s yellow) and current injection (60 pA, 3 s) meant to mimic synaptic inputs. Note the efficient silencing of firing even in the presence of a depolarizing current pulse. EGFP, enhanced green fluorescent protein; MC, mitral cell; OB, olfactory bulb; P, postnatal day; RMP, resting membrane potential. (TIF) [file pbio.2006994.s005.tif]

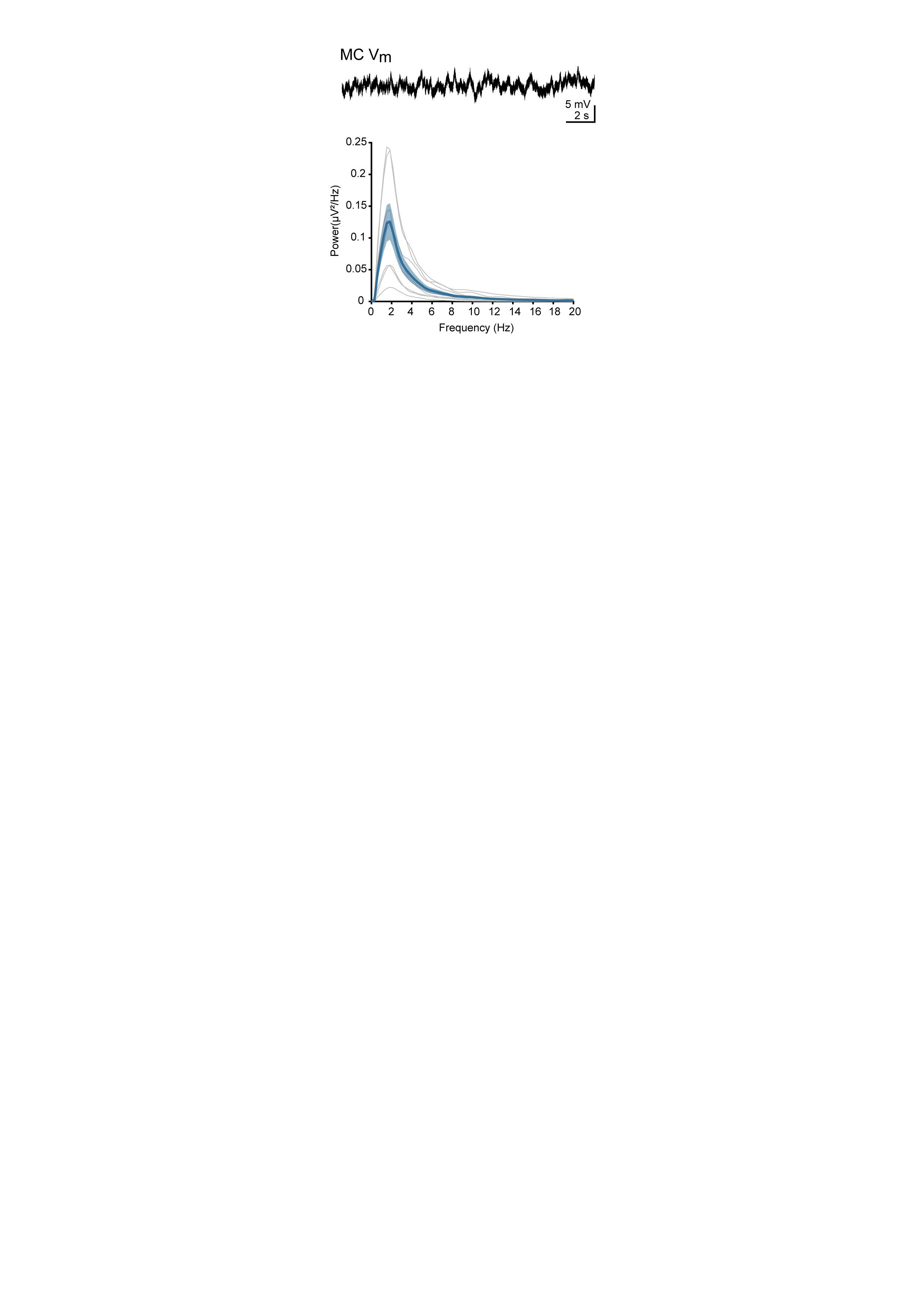

Supplement: S6 Fig — Membrane potential oscillations of MCs in vitro. Top, representative current-clamp recording from a P9 MC (−48 mV) showing membrane potential oscillations. Bottom, power spectrum (blue, mean ± SEM) of membrane voltage oscillations of MCs with firing rates < 0.1 Hz (blue). Power spectra of individual MCs are shown in gray. MC, mitral cell. (TIF) [file pbio.2006994.s006.tif]

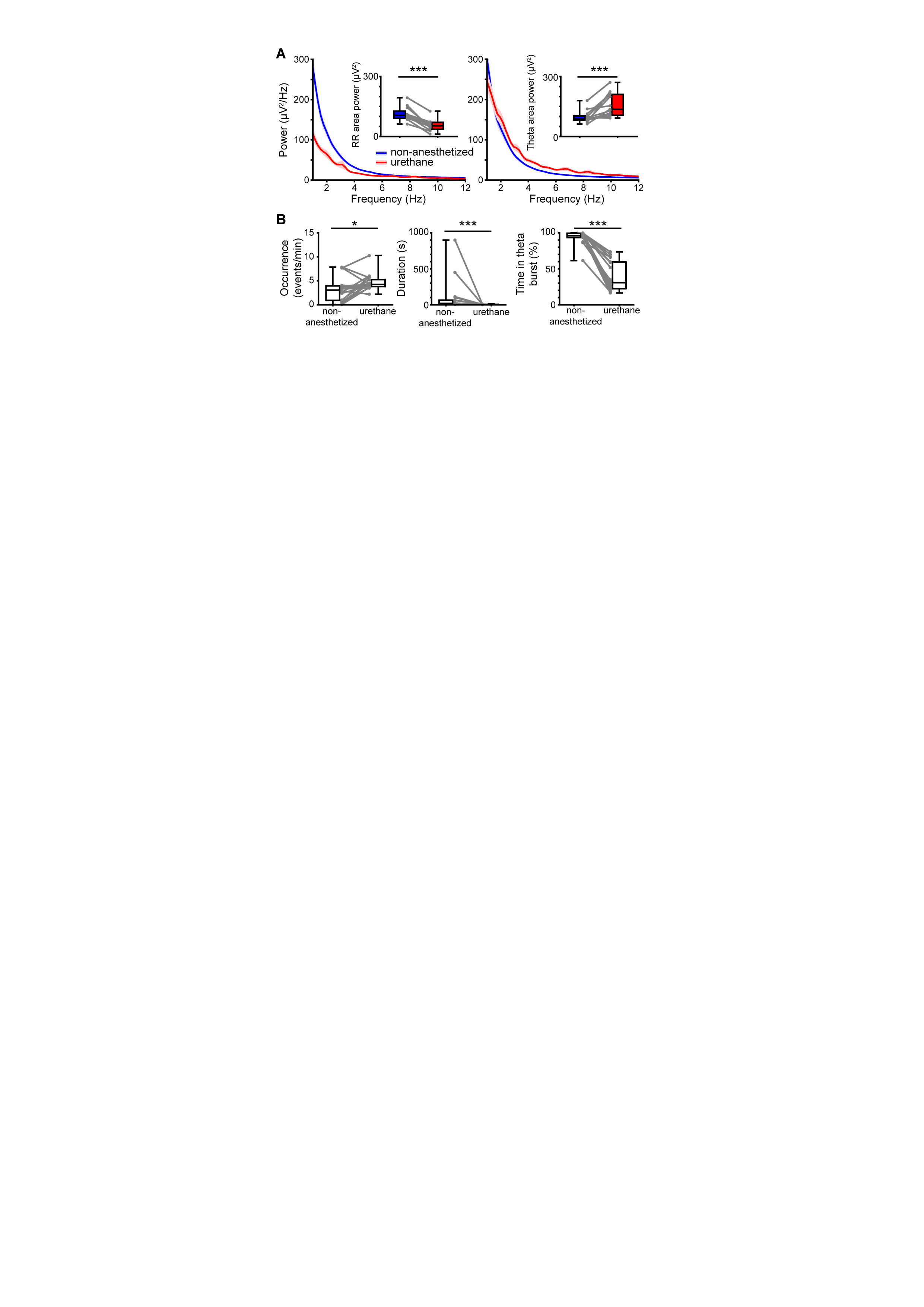

Supplement: S7 Fig — Effects of urethane anesthesia on the network activity in neonatal LEC. (A) Power spectra (mean ± SEM) of LFP recorded in the neonatal LEC before (blue) and during (red) urethane anesthesia when calculated for the entire trace (left) and for concatenated time windows of theta bursts (right). Insets, box plots displaying RR and theta power before and during urethane anesthesia (n = 12, 1 outlier removed, n = 13). (B) Box plots displaying the occurrence and duration of theta bursts as well as the level of discontinuity of theta bursts measured as a fraction of recording time with activity in theta band in neonatal LEC (n = 18). Gray dots and lines correspond to individual animals. (*p < 0.05; ***p < 0.001; Wilcoxon signed-rank test). Data are available in S1 Data. LEC, lateral entorhinal cortex; LFP, local field potential; RR, respiration-related rhythm. (TIF) [file pbio.2006994.s007.tif]

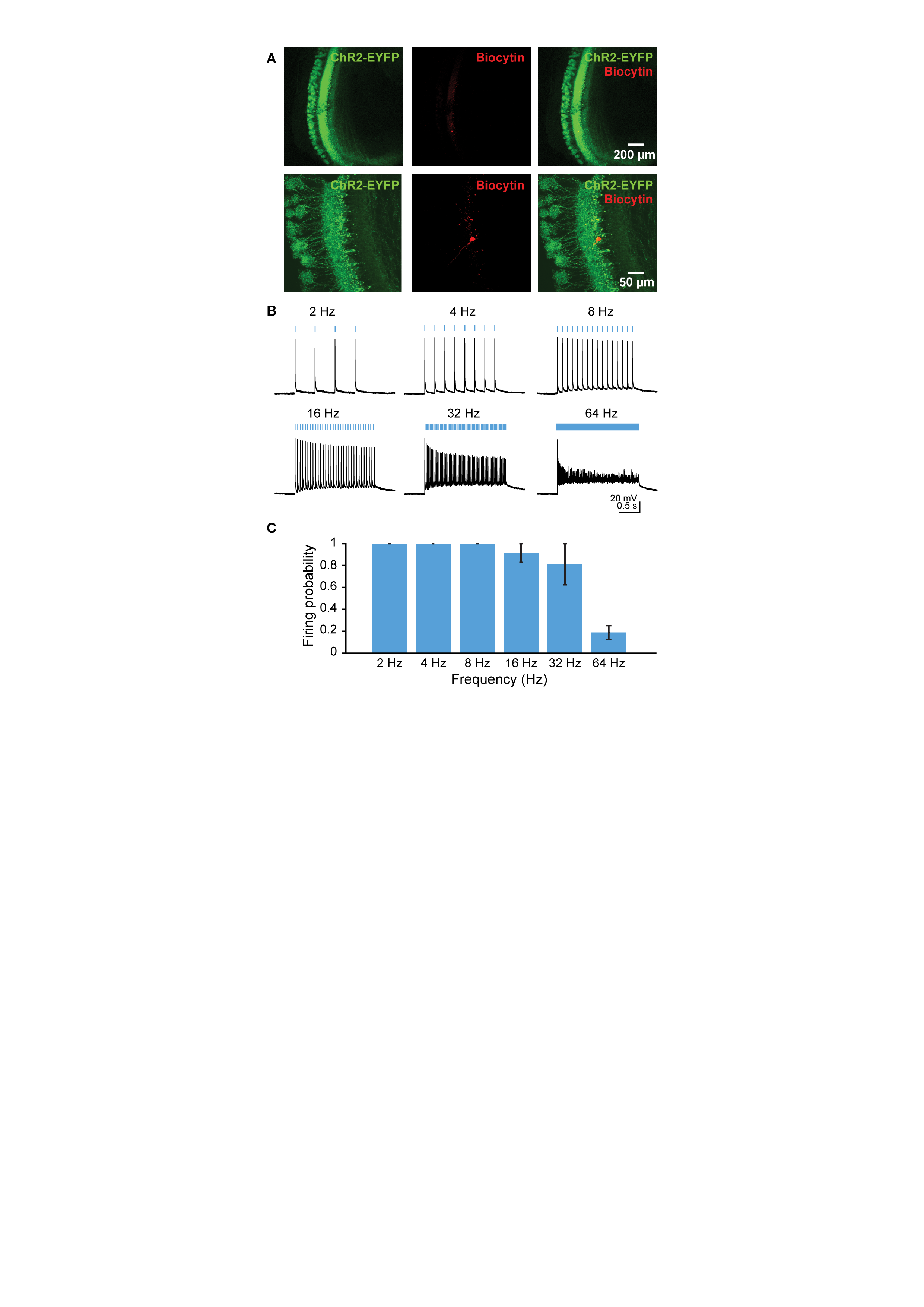

Supplement: S8 Fig — Optogenetic activation of MCs in vitro. (A) Top, digital photomontage reconstructing by high-resolution confocal imaging the morphology of a biocytin-filled (red), EYFP-stained (green) ChR2-positive MC in the OB of a P6 mouse. Bottom, same MC displayed at higher magnification. (B) Representative voltage responses of a transfected MC to trains of 3 ms–long light stimuli at different frequencies. (C) Bar diagram displaying the mean firing probability of transfected neurons in response to repetitive light stimulation at different frequencies (n = 4 neurons). Data are available in S1 Data. ChR2, channelrhodopsin 2; EYFP, enhanced yellow fluorescent protein; MC, mitral cell; OB, olfactory bulb; P, postnatal day. (TIF) [file pbio.2006994.s008.tif]

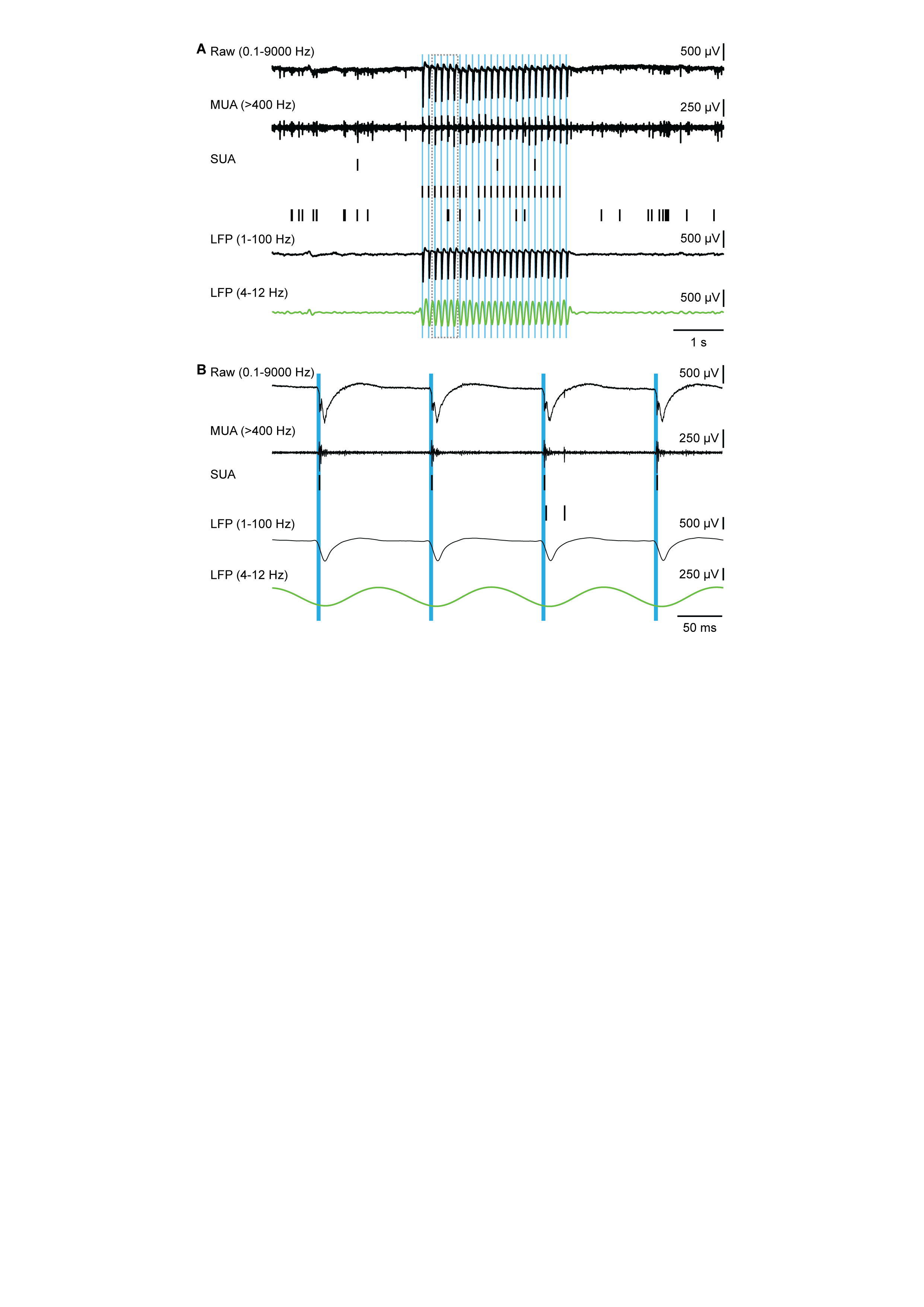

Supplement: S9 Fig — Light-evoked spike responses of MTC in relationship to network oscillations. (A) Raw signal, band-pass-filtered LFP (1–100 Hz, theta 4–12 Hz), MUA (<400 Hz), and SUA spike trains before, during, and after pulsed (8 Hz) light stimulation (473 nm) of MTCs in OB of a P8 Cre+ Tbet-cre mouse. (B) Same as (A, dotted box) displayed at larger timescale. LFP, local field potential; MTC, mitral and tufted cell; MUA, multiunit activity; OB, olfactory bulb; P, postnatal day; SUA, single-unit activity. (TIF) [file pbio.2006994.s009.tif]

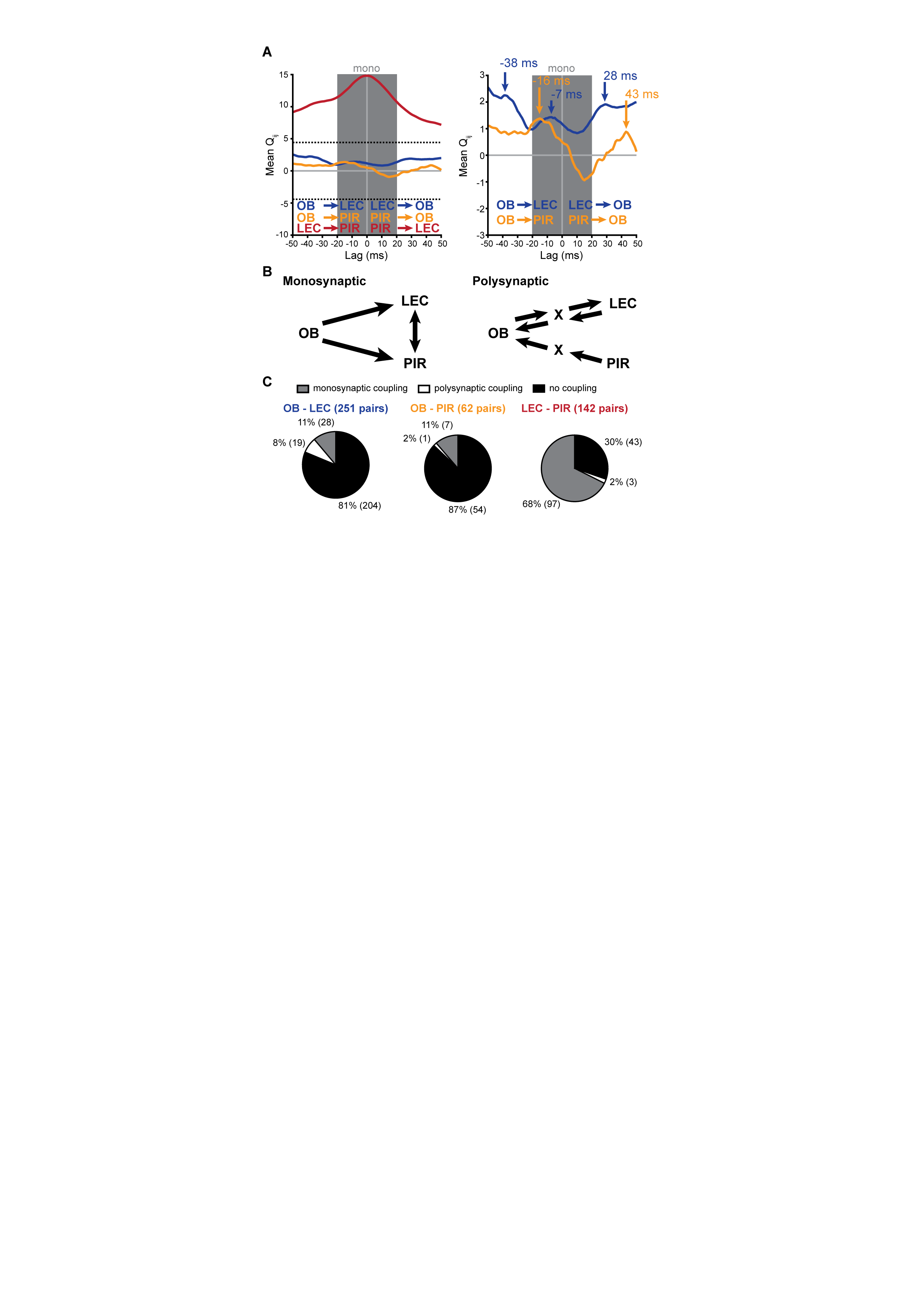

Supplement: S10 Fig — (A) Left, line plots showing smoothed mean standardized cross-covariance of spike pairs recorded from OB and LEC (n = 251), OB and PIR (n = 62), and LEC and PIR (n = 142) that have significant coupling at lags between −50 and 50 ms (black dotted lines correspond to significance threshold). Right, same plot displayed at higher magnification to highlight the cross-covariance peaks for OB–LEC and OB–PIR spike trains. (B) Schematic overview of mono- and polysynaptic connectivity between OB, LEC, and PIR as revealed by cross-covariance analysis of spike trains. (C) Pie charts showing the percentage of monosynaptically coupled, polysynaptically coupled, and uncoupled unit pairs. LEC, lateral entorhinal cortex; OB, olfactory bulb; PIR, piriform cortex. (TIF) [file pbio.2006994.s010.tif]
