# Supplementary material for: Coordinated electrical activity in the olfactory bulb gates the oscillatory entrainment of entorhinal networks in neonatal mice
Source: PLoS Biol. 2019 Jan 31;17(1):e2006994. doi: 10.1371/journal.pbio.2006994 (PMC6354964; doi:10.1371/journal.pbio.2006994)
Supplement: S1 Table — Effect of urethane anesthesia on activity patterns in neonatal OB and LEC. The values are given as medians and interquartile ranges, and significant differences are shown as *p < 0.05, ***p < 0.001 (Wilcoxon signed-rank test). LEC, lateral entorhinal cortex; OB, olfactory bulb. (DOCX) [file pbio.2006994.s011.docx]

|  | | OB | | | | | | | LEC | | | | | | |
| --- | --- | --- | --- | --- | --- | --- | --- | --- | --- | --- | --- | --- | --- | --- | --- |
|  | | **Non-anesthetized** | | **Urethane-anesthetized** | | p | | **Non-anesthetized** | | | **Urethane-anesthetized** | | p |  |  |
| RR area power  (µV^2^) | 113.00  69.88-134.96 | | 60.21  44.36-109.41 | | 0.0771 | | 105.62  90.88-126.84 | | | 53.42  36.38-70.90 | | **0.0005**  **(***)** | | |  |
| Theta burst area power (µV^2^) | 110.60  80.91-148.84 | | 155.71  125.65-195.70 | | **0.0161**  **(*)** | | 88.57  82.37-102.85 | | | 136.60  106.63-210.60 | | **0.0005**  **(***)** | | |  |
| Theta burst occurrence  (events/min) | 0.13  0.07-5.67 | | 4.20  3.40-6.60 | | 0.1024 | | 3.07  0.93-3.93 | | | 4.23  3.80-5.27 | | **0.0198**  **(*)** | | |  |
| Theta burst duration (s) | 449.77  5.12-899.80 | | 3.62  2.69-4.38 | | **0.0002**  **(***)** | | 18.63  14.27-63.89 | | | 4.34  3.57-6.17 | | **0.0002**  **(***)** | | |  |
| Time in theta burst (%) | 99.95  77.97-99.98 | | 26.46  19.45-35.10 | | **0.0002**  **(***)** | | 96.14  93.56-99.42 | | | 30.82  22.35-59.66 | | **0.0002**  **(***)** | | |  |
