# Supplementary material for: Coordinated electrical activity in the olfactory bulb gates the oscillatory entrainment of entorhinal networks in neonatal mice
Source: PLoS Biol. 2019 Jan 31;17(1):e2006994. doi: 10.1371/journal.pbio.2006994 (PMC6354964; doi:10.1371/journal.pbio.2006994)
Supplement: S1 Text — (DOCX) [file pbio.2006994.s013.docx]

**Supporting Methods**

**Experimental model and subject details**

*Mice.* Timed-pregnant C57Bl/6J and Tbet-cre mice from the animal facility of the University Medical Center Hamburg-Eppendorf as well as B6.Cg-*Gt(ROSA)26Sor^tm40.1(CAG-aop3/EGFP)Hze^*/J mice (Ai40(RCL-ArchT-EGFP)-D, Jackson Laboratory, stock no: 02118), and Tbet-cre;ArchT-EGFP mice (bred by the animal facility of the University Medical Center Hamburg-Eppendorf) were housed individually in breeding cages at a 12 h light / 12 h dark cycle and fed *ad libitum*. Mouse lines used for CLARITY experiments (Tbet-cre mice, B6.Cg-*Gt(ROSA)26Sor^tm9(CAG-tdTomato)Hze^*/J (Ai9(RCL-tdT), Jackson Laboratory, stock no: 007909 and Tbet-cre;tdT mice) were bred in the animal facility at RWTH Aachen University under similar conditions. The day of vaginal plug detection was defined E0.5, while the day of birth was assigned as P0. Male mice underwent sensory manipulation, light stimulation, pharmacological treatment and multi-site electrophysiological recordings at P8-10. For CLARITY experiments, male and female mice were used. Genotypes were determined using genomic DNA and following primer sequence (Metabion, Planegg/Steinkirchen, Germany: for Cre in Ai40(RCL-ArchT-EGFP)-D mice: PCR forward primer 5’-ATCCGAAAAGAAAACGTTGA-3’ and reverse primer 5’-ATCCAGGTTACGGATATAGT-3’; for ROSA26-wt PCR forward primer 5’-AAAGTCGCTCTGAGTTGTTAT-3’ and reverse primer 5’-GGAGCGGGAGAAATGGATATG-3’; for GFP-tg PCR forward primer 5’-CTGGTCGAGCTGGACGGCGACG-3’ and reverse primer 5’-GTAGGTCAGGGTGGTCACGAG-3’; for Cre in Ai9(RCL-tdT) mice: forward primer 5’ CATGTCCATCAGGTTCTTGC 3’ and reverse primer 5’ AGAGAAAGCCCAGGAGCAG 3’; for tdTomato forward primer 5’ GGCATTAAAGCAGCGTATCC 3’ and reverse primer 5’ CTGTTCCTGTACGGCATGG 3’. The PCR reactions were as follows: 10 min at 95°C, 30 cycles of 45 s at 95°C, 90 s at 54°C, 90 s at 72°C, followed by a final extension step of 10 min at 72°C (Cre-tg and ROSA26-wt), 10 min at 95°C, 30 cycles of 45 s at 95°C, 90 s at 68°C, 90 s at 72°C, followed by a final extension step of 10 min at 72°C (GFP-tg). In addition to genotyping, EGFP expression in OB prior to surgery was detected using a dual fluorescent protein flashlight (Electron microscopy sciences, PA, US).

**Methods details**

***Surgical procedures****Surgical preparation for electrophysiology and light delivery in vitro.* For patch-clamp recordings, pups were decapitated and brains were sliced with a vibroslicer (Leica, VT1000S) in 300 μm-thick coronal sections in ice-cold oxygenated high sucrose-based artificial cerebral spinal fluid (ACSF) containing (in mM) 228 sucrose, 2.5 KCl, 1 NaH_2_PO_4_, 26.2 NaHCO_3_, 11 glucose, 7 MgSO_4_ (320 mOsm). Slices were incubated in oxygenated ACSF containing (in mM) 119 NaCl, 2.5 KCl, 1 NaH_2_PO_4_, 26.2 NaHCO_3_, 11 glucose, 1.3 MgSO_4_ (320 mOsm) at 37 °C. Prior to recordings, slices were maintained at room temperature and superfused with oxygenated ACSF.

*Surgical preparation for electrophysiology and light delivery in vivo.* For recordings in non-anesthetized state, 0.5% bupivacain / 1% lidocaine was locally applied on the neck muscles. For recordings under anesthesia, mice were injected i.p. with urethane (1 mg/g body weight; Sigma-Aldrich, MO, USA) prior to surgery. For both groups, under isoflurane anesthesia (induction: 5%, maintenance: 2.5%) the head of the pup was fixed into a stereotaxic apparatus using two plastic bars mounted on the nasal and occipital bones with dental cement. The bone above the right OB (0-5-0.8 mm anterior to fronto-nasal suture, 0.5 mm lateral to inter-nasal suture) as well as LEC (0 mm posterior to lambda, 6-6.5 mm lateral from the midline) was carefully removed by drilling a hole of <0.5 mm in diameter. Throughout surgery and recording session the mice were maintained on a heating blanket at 37°C.

*Virus injection for transfection of MTCs with ChR2.*

For transfection of mitral cells with ChR2 mutant (E123T/T159C), P0-1 pups were fixed into stereotaxic apparatus and received unilateral injections of the viral construct (pAAV-Ef1a-DIO hChR2(E123T/T159C)-EYFP, 100 µl at titer ≥ 1×10¹³ vg/mL, Addgene, MA, USA) into right OB. A total volume of 200-400 nl was slowly (200-400 nl/min) delivered at a depth of around 0.5 mm into OB using a micropump (Micro4, WPI, Sarasota, FL). Following injection, the syringe was left in place for at least 30 s to avoid reflux of fluid. The surgical openings were sealed with fibrin glue (Surgibond, SMI Sutures). Pups were maintained on a heating blanket until full recovery and returned to the dam.

***Electrophysiology****Electrophysiological recordings in vivo.* One-shank electrodes (NeuroNexus, MI, USA) with 16 recording sites (0.4-0.8 MΩ impedance, 50 µm inter-site spacing for OB, 100 µm inter-site spacing for LEC) were inserted into dorsal (depth 0.5-1.2 mm, angle 0°) or ventral OB (1.4-1.8 mm, angle 0°) as well as in LEC (depth: 2 mm, angle: 10° from the vertical plane). Two-shank optoelectrodes (Buzsaki16-OA16LP, NeuroNexus, MI, USA) with 8 recordings sites on each shank (1-4 MΩ impedance, 20-40 µm inter-site spacing, 200 µm inter-shank spacing) aligned with an optical fiber (50/62.5 µm (etched) diameter) ending 40 μm above the top recording site were inserted into ventral OB. Before insertion, the electrodes were covered with DiI (1,1’-Dioctadecyl-3,3,3’,3’-tetramethylindocarbocyanine perchlorate, Molecular Probes, Eugene, OR). A silver wire was inserted into the cerebellum and served as ground and reference electrode. Before data acquisition, a recovery period of 20 min following the insertion of electrodes was provided. Extracellular signals were band-pass filtered (0.1 Hz – 9 kHz) and digitized (32 kHz) by a multichannel amplifier (Digital Lynx SX; Neuralynx, Bozeman, MO; USA) and Cheetah acquisition software (Neuralynx). The signal from the piezo-electric movement detector that was used for assessment of respiration was filtered (<48 Hz) online to remove noise. Spontaneous (i.e. not induced by manipulation or stimuli) activity was recorded for 15 min at the beginning and end of each recording session and considered baseline activity. The position of recording electrodes in OB and LEC was confirmed after histological assessment *post-mortem*: For the analysis of LFP in OB, the recording site centered in external plexiform layer was used, whereas for the analysis of spiking activity recording sites located in the mitral cell layer (50 µm above and below on the linear probe) were considered. Spike time overlap between pairs of units from different sites was below 4%. When recordings were done with two-shank optoelectrodes, only one recording site per shank was used for spike analysis to ensure that detected units stem from different cells. For the analysis of LFP and spiking activity in LEC, only recording sites that were histologically confirmed to be located in LEC were used for analysis. The LFP activity was analyzed at recording sites located in superficial layers, whereas the spiking activity was quantified at all recording sites confined to LEC.

*Electrophysiological recordings in vitro.* Whole-cell patch-clamp recordings were performed from MTCs identified by their location in the mitral cell layer and visualized by membrane-bound EGFP. All recordings were performed at room temperature. Recording electrodes (4-9 MΩ) were filled with K-gluconate based solution containing (in mM): 130 K-gluconate, 10 HEPES, 0.5 EGTA, 4 Mg-ATP, 0.3 Na-GTP, 8 NaCl (285 mOsm, pH 7.4) and 0.5% biocytin for post-hoc morphological identification of recorded cells. Recordings were controlled with the Ephus software [1] in the MATLAB environment (The MathWorks, Inc., MA, USA). Capacitance artefacts were minimized using the built-in circuitry of the patch-clamp amplifier (Axopatch 200B; Molecular devices, CA, USA). The signals were low-pass filtered at 10 kHz and recorded online. All potentials were corrected for the liquid junction potential of the gluconate-based electrode solution, which according to our measurement was -8.65 mV. The resting membrane potential (RMP) was measured immediately after obtaining the whole-cell configuration. For the determination of input resistance (R_in_), membrane time constant (τ_m_) and membrane capacitance (C_m_) hyperpolarizing current pulses (-60 pA) of 600 ms duration were applied from resting membrane potential. Spontaneous membrane oscillations were analyzed offline using custom-written scripts in the MATLAB environment (Version 9, MathWorks, Natick, MA). Data were band-passed (1-48 Hz) filtered using a third-order Butterworth filter before down-sampling by factor 10 to 1 kHz. Only neurons with firing rate < 0.1 Hz were considered.

***Morphological investigation***

*CLARITY.* Neonatal mice of both sexes were sacrificed by decapitation. Brains were sliced with a vibroslicer (Leica VT1000S) in 1 mm- (for LEC) and 500 µm-thick (for OB) coronal sections. To maintain the structural integrity, the tissue was fixed overnight at 4°C in hydrogel fixation solution containing 4% acrylamide, 0.05% bis-acrylamide, 0.25% VA-044 Initiator, 4% PFA in PBS-/-. To allow hydrogel formation, oxygen-free conditions were ensured by using a vacuum pump connected to a desiccator and argon environment. After 3 h of incubation at 37 °C for polymerization, samples were extracted from hydrogel and washed in clearing solution containing 200 mM boric acid and 138 mM SDS (pH 8.5) for 24 h at RT. Afterwards, they were embedded and maintained in clearing solution at 37 °C for 7 to 10 days. The nuclear marker DRAQ5 (1:1000) was added to the samples for at least 2 days before three washing steps with PBST (0.1% TritonX in PBS^-/-^) at RT finished the clearing procedure by removal of SDS. Subsequently, the samples were incubated for 24 h in RIMS80 containing 80 g Nycodenz, 20 mM PS, 0.1% Tween 20, and 0.01% sodium acid.

*Retrograde tracing.* For retrograde tracing, anesthetized P3-4 mice were fixed into stereotaxic apparatus and received unilateral Fluorogold (FG) (Fluorochrome, LLC, USA) injections into OB (0.8 mm anterior from the fronto-nasal suture, 0.8 mm from midline) or LEC (0 mm posterior to bregma, 5 mm from midline). A total volume of 50-600 nl for OB and 30-50 nl for LEC was slowly (100-800 nl/min) delivered at a depth of 0.5-1 mm (OB) and 0.2 mm (LEC) using a micropump (Micro4, WPI, Sarasota, FL). Following injection, the syringe was left in place for at least 2 min to avoid reflux of fluid. The surgical openings were sealed with fibrin glue (Surgibond, SMI Sutures). Pups were maintained on a heating blanket until full recovery and returned to the dam. After 4-5 days, pups were deeply anesthetized and perfused at P8.

*Perfusion.* Mice were anesthetized with 10% ketamine (aniMedica, Germany) / 2% xylazine (WDT, Germany) in 0.9% NaCl solution (10 µg/g body weight, i.p.) and transcardially perfused with Histofix (Carl Roth, Germany) containing 4% paraformaldehyde (PFA). Brains were postfixed in 4% paraformaldehyde for 24 h.

*Staining protocols*. Mice were terminally anesthetized with an i.p. injection of 10% ketamine (aniMedica, Germany) / 2% xylazine (WDT, Germany) in 0.9% NaCl (B. Braun, Melsungen, Germany) solution (10 µg/g body weight) and transcardially perfused and post-fixed with 4% paraformaldehyde (Carl Roth, Germany). For fluorescent Nissl or bisBenzimide stainings, mounted 100 µm-thick sections were rehydrated in PBS and washed in PBST (0.2% Triton) and PBS. Sections were incubated for 20 min either with 200 µl of NeuroTrace Fluorescent Nissl stain (1:100 in PBS; Molecular Probes, Eugene, OR, USA) or 200 µl bisBenzimide (5 µg/ml, Sigma-Aldrich) in a humid chamber. Sections were washed in PBST and PBS and mounted with Fluoromount (Sigma-Aldrich). Brain slices used for *in vitro* recordings were fixed in 4% PFA, washed in PBS, blocked and permeabilized (0.8% Triton, Sigma-Aldrich, 5,0% normal bovine serum albumin, Jackson Immuno Research, 0.05% sodium azide, Sigma-Aldrich). Staining with Cyanine dye 2 (Cy2)-conjugated streptavividin (Jackson Immuno Research, 1:400 in PBS, with 3% bovine serum albumin, Jackson Immuno Research and 0.05% sodium azide, Sigma-Aldrich) for 60 min was used to identify biocytin-filled cells. After washing in PBS, slices where mounted with Fluoromount. For GABA staining, 50 µm-thick coronal brain sections containing the LEC were treated with PBS containing 0.2% Triton-X100 (Sigma-Aldrich), 10% normal bovine serum (Jackson Immuno Research) and 10% donkey serum (Millipore). Sections were incubated overnight at 4°C with rabbit polyclonal primary antibody against GABA (gamma-aminobutyric acid) (1:1000, #A2053, Sigma-Aldrich) or CamKII (1:200, PA5-38239, Thermo Fisher Scientific, MA, USA) and Alexa Fluor-568 donkey anti-rabbit IgG secondary antibody (1:1000, A10042, Invitrogen).

*Microscopy and image acquisition.* Wide-field fluorescence images were acquired to reconstruct the position of the DiI-labeled recording electrodes in brain slices of investigated pups and to take overview images for retrograde tracing experiments. Photographs were taken using an Olympus SZX16 microscope and an Olympus DP72 camera. Overlay images were created using Olympus cellSens Software, ImageJ or Photoshop. Magnification images of fluorogold-labeled cell bodies were acquired using a Zeiss Axio scope 2 MOT and a Zeiss AxioXam HRc. Close-up images of EGFP expression in OB and LEC as well as of biocytin-stained cells were acquired on a confocal microscope (DM IRBE, Leica, Germany). For CLARITIY experiments, imaging was performed with Leica DM 6000 CFS microscope, a CLARITY-optimized HC FLUOTAR L 25x/1.00 IMM objective, and a cooled CCD camera (DFC365 FX; Leica Microsystems). Confocal images of cleared samples were acquired sequentially exciting with a 638 nm laser for DRAQ5 and a 552 nm laser for tdTomato using a Hybrid detector (Leica Microsystems).

***Manipulations***

*Light stimulation in vitro.* Whole-cell current-clamp recordings were performed from ArchT-EGFP or ChR2-EYFP expressing mitral cells in coronal slices of the neonatal Tbet-cre;ArchT mice or Tbet-cre mice transfected with a cre-dependent virus carrying ChR2. Yellow light pulses (595 nm) of different light intensities (1.5-19.3 mW mm^-2^) were applied for 5 s (ramp up 1 s, 3 s constant light intensity, ramp down 1 s) at resting membrane potential to test the effect on the membrane potential. During spontaneous action potential firing light pulses of 0.77 mW were repeatedly applied for 5 min (pulse duration 5 s, inter-pulse interval 10 s). Light stimulation (intensity: ~0.77 mW) was paired with current injections of 20 to 60 pA. Trains of blue light pulses (470 nm, 3 ms, 7.2-50.7 mW mm^-2^) with different frequencies were applied at resting membrane potential to induce action potential firing in ChR2-transfected mitral cells.

*Light stimulation in vivo.* Inhibition of MTC activity was achieved by trapezoid light stimulation applied using a diode pumped solid state (DPSS) laser (Cobolt Mambo, 594 nm, Omicron, Austria). Resulting laser power was 300 mW/mm^2^ at the fiber tip of optoelectrode. No light artifacts were observed. A trapezoid-shaped light stimulus with 1 s rise time, 10 s constant period, 1 s fall time, and a 48 s inter-stimulus interval was presented 30 times. For activation of MTCs, pulsed (laser on-off) light stimulations (3ms) were performed with a diode laser (473 nm; Omicron, Austria). Resulting light power on was in the range of 38.2–103.8 mW mm^-2^ at the fibre tip. Both lasers where controlled with an arduino uno (Arduino, Italy).

*Estimation of light propagation*. The spatial pattern of light propagation *in vivo* was estimated using a recently developed model [2] based on Monte Carlo simulation (probe parameters: light fibre diameter: 50 µm, numerical aperture: 0.22, light parameters: 594 nm, 0.6 mW).

*Naris occlusion.* One naris was closed using silicon adhesive (Kwik-Sil, World Precision Instruments). After a recovery period of five minutes, the recording was pursued while one naris was sealed.

*Pharmacological inactivation.* To block the firing of OB neurons, lidocaine hydrochloride (Sigma-Aldrich, 4 µl, 4% in 0.9% NaCl, pH 7.0 with NaOH) was slowly (800 nl/min) infused into the OB (1 mm depth) using a 10 µl Hamilton syringe attached to a micropump. The volume of lidocaine was calculated to ensure inactivation of 90% of OB neurons according to the spherical volume equation [3]. The confinement of lidocaine to OB was confirmed by injection of 4 µl methylene blue (Sigma-Aldrich) into OB using the same coordinates. For analysis, LFP and MUA during baseline conditions (30 min before injection of lidocaine) were compared with the activity recorded during the first 30 min after lidocaine application.

*Lesion of nasal epithelium.* Methimazole (Sigma-Aldrich, 100 mg/kg in sterile saline) or saline was injected intraperitoneally at P3. Extracellular recordings of LFP and MUA were performed in P8-10 controls (i.e. saline-treated) and methimazole-treated pups. Monitoring of weight from P3 to P8 showed that methimazole-treated pups (n=11) gained less weight when compared to their saline-treated littermates (n=13) (p=6.40 x10^-5^, Wilcoxon rank-sum test, saline: median: 108.61, iqr: 89.02-120.04; methimazole: median: 52.51, iqr: 30.64-78.85). This effect might be due to the difficulties of pups with a degenerated nasal epithelium to find dam’s nipple for feeding.

*Odor stimulation.* An eight channel dilution olfactometer (Aurora Scientific) was used for stimulus delivery. Clean air was passed through an activated charcoal filter and separated into a stream of clean air and a stream passing through one of the odor vials. A continuous stream of clean air was delivered to the animal’s nose. During stimulus presentation, odorized air was mixed with clean air (40:60) and presented at a flow rate of 200 sccm. Each of the stimuli was presented 8-10 times for 2 s with a 30 s gap (randomized order). Vacuum removal of odors after stimulus presentation was ensured.

**Quantification and statistical analysis**

*Immunohistochemistry quantification.* Images were analyzed using ImageJ. To quantify FG-labeled and double (FG and GABA)-labeled cell bodies, manual cell counting was performed using ImageJ (cell counting plug in).

*Detection of respiration frequency.* Respiration was monitored using a piezo-electric sensor placed under the pup’s chest. Deflections were detected using MATLAB ‘findpeaks’ function on 5 minute long data segments. Large negative peaks marked the transition from inhalation to exhalation.

*LFP analysis*. Data were analyzed offline using custom-written scripts in the MATLAB environment (Version 9, MathWorks, Natick, MA). Data were first low-passed filtered (<100 Hz) using a third-order Butterworth filter before downsampling by factor 20 to 1.6 kHz to analyze LFP. All filtering procedures were performed in a manner preserving phase information.

*Detection of oscillatory activity.* Discontinuous network oscillations in the LFP recorded from OB and LEC were detected using a previously developed unsupervised algorithm [4]. Briefly, deflections of the root mean square of band-pass filtered (1-100 Hz) signals exceeding a variance-depending threshold (2.5 times the standard-deviation from the mean) were assigned as oscillatory periods. Only oscillatory periods lasting at least 1 s were considered for analysis.

*Power spectral density.* Power spectral density was calculated using Welch’s method. Oscillatory periods (for theta), entire signal (for RR) or periods of light stimulation were divided into segments of 3 s length and concatenated. The power spectra of resulting data were calculated for an fft length of 2^13^ and were used to determine the area power for specific frequency bands using trapezoidal numerical integration. Time-frequency plots of power were calculated with a continuous wavelet transform (Morlet wavelet).

*Coherence*. The imaginary part of coherence, which is insensitive to volume-conduction-based effects [5], was calculated by taking the absolute value of the imaginary component of the normalized cross-spectrum:

$$C_{XY}(f)=\left| Im\left( \frac{P_{XY}\left( f \right)}{\sqrt{{P_{XX}(f)P}_{YY}(f)}} \right) \right|$$

Frequency domains with significant coherence were determined by Monte Carlo simulation*.* For this, the LFP segments from one region were shuffled with respect to data from the other region and the coherence was calculated. Shuffling was performed 1000 times and the 95^th^ percentile of the resulting distribution was used as significance threshold.

*Inter-regional LFP phase difference.* Frequency-dependent phase differences between OB and LEC were calculated as follows: First, signals from both regions were bandpass filtered (2-4 Hz, 4-12 Hz) using a third-order Butterworth filter preserving phase information. Subsequently, a Hilbert transform was applied to filtered signals to obtain the instantaneous phase. Only phase information extracted during simultaneous theta bursts was used. The LEC phase was subtracted from the OB phase, which means that positive values correspond to LEC preceding OB signal.

The optogenetic modulation index (OMI) was calculated as

$$\frac{{Power}_{during}-{Power}_{pre}}{{Power}_{during}+{Power}_{pre}}.$$

*Spike sorting*. Spike sorting was performed offline based on waveform shape using spike sorting software (Plexon). The raw signal was high-pass filtered (>400 Hz) and the threshold for spike detection was set individually at around -6 standard deviations from the mean. A group of similar waveforms was considered as being generated from a single neuron if it defined a discrete cluster in a 2D/3D space and exhibited a clear refractory period (>1 ms) in the interspike interval histogram. The quality of separation between identified clusters was assessed by four different statistical measurements: the classical parametric F statistic of multivariate analysis of variance (MANOVA), the J3 and PseudoF (Psf) statistics and the Davies-Bouldin validity index (DB) [6,7]. The values of statistical testing in OB ranged between 4.45x10^-10^ and 0.17 for MANOVA, 0.4 and 14.24 for J3, 360.63 and 61876.9 for PsF, and 0.26 and 0.69 for DB, whereas in LEC they ranged between 3.70x10^-11^ and 0.07 for MANOVA, 0.85 and 6.80 for J3, 401.76 and 9332.15 for PsF, and 0.14 and 0.46 for DB. A total number of 122 units were clustered in OB and 254 in LEC. One to four units per recording site were identified.

*Spike-LFP coupling*. Intra- and inter-regional phase locking of spiking of clustered units to network oscillations was assessed using a previously described algorithm [8,9]. For this, the raw LFP signal was bandpass filtered (2-4 Hz, 4-12 Hz) using a third-order Butterworth filter preserving phase information. Subsequently, a Hilbert transform was applied to the filtered signal to obtain the instantaneous phase. If the firing of a neuron was modulated by oscillations within a specific frequency band, then its phase over the oscillatory cycle should be not uniformly distributed. Phases of π referred to the trough and phases of 0/2π referred to the peak of the cycle. The coupling between spikes and network oscillations was tested for significance using the Rayleigh test for non-uniformity. The spike trains were converted into a sequence of unit length vectors oriented by the phase of their corresponding spikes. The value of Rayleigh’s *R* statistic indicates strength of phase coupling (or degree of non-uniformity) between unit events and field potential and was computed as

$$R=nr$$

Where *r* denotes the mean resultant vector (MRV) length of the given phase series and *n* is the number of phase values. The probability that the null hypothesis of sample uniformity holds is given by

$$P= e^{\sqrt{1+4n+4\left( n^{2}-R^{2} \right)}-(1+2n)}$$

Only neurons that showed significant phase locking were considered for analysis of preferred phase. To compare distributions of preferred phases, Kuiper’s two-sample test was used. Locking strength was calculated as MRV length. When assessing the locking to RR, all detected spike were used, where as when assessing the locking to theta band activity, only spikes occurring during theta bursts were used. Whenever a comparison between groups with different spike numbers was performed, either phase locking was calculated using a fixed spike number for both groups, or pair-wise phase consistency, a spike number-independent measure [10], was used. For this, pairwise phase consistency was calculated as

$$PPC=\frac{2}{N(N-1)}\sum_{j=1}^{N-1} \sum_{k=j+1}^{N} cos(d(\theta_{j},\theta_{k}))$$

where, θ_j_, θk are the phases of LFP samples assigned to the contemporaneous spikes, *N* is the number of spikes, and d(ϕ,ω) is the absolute angular distance defined as the function

$$d\left( \varphi,\omega\right)=\left| \varphi- \omega\right| mod \pi$$

PPC=1 indicates complete phase consistency, whereas PPC=0 indicates lack of phase locking.

For phase-locking analysis during light stimulation of hChR2-transfected neurons, only units with at least 25 spikes during the total stimulation time were used. To exclude effects due to firing rate differences, a fixed number of 25 spikes was used for all units and phase-locking was calculated. This procedure was repeated 1000 times and the median of the resultant vector length and of the preferred phase was used. To assess statistical significance of phase-locking, a unit was considered as being significantly locked if more than 95% of the calculated p-values were significant.

*Cross-covariance of spike trains.* The analysis of timing between pairs of simultaneously recorded spike trains form OB and LEC, PIR and LEC, as well as OB and PIR was performed as previously described [9]. For two spike trains $N_{i}$ and $N_{j}$, the cross-covariance estimate was calculated as

$\hat{q}_{ij}\left( u \right)=\frac{J_{ij}^{T,b}(u)}{bT}-\hat{P}_{i}\hat{P}_{j}$,

where $J_{ij}^{T,b}(u)$ is the cross-correlation histogram with bin size $b$and observation period $T$ and $\hat{P}_{i}=\frac{N_{i}(T)}{T}$*,* $\hat{P}_{j}=\frac{N_{j}(T)}{T}$. The standardized cross-covariance was calculated as

$Q_{ij}\left( u \right)=\sqrt{\frac{bT}{P_{i}P_{j}}}\hat{q}_{ij}(u)$*,*

with $P_{i},P_{j}$ being the mean firing rates. Only pairs of units with firing rates > 0.05 Hz were considered.

*Statistics.* Statistical analysis was performed using SPSS Statistics 22 (IBM, NY) or MATLAB. Gaussian distribution of the data was assessed using the Kolmogorov-Smirnov test for sample size >10. None of the data sets were normally distributed. Therefore, data were tested for significance using Wilcoxon signed-rank test (2 related samples), Wilcoxon rank-sum test (2 unrelated samples), Friedman test (>2 related samples; Wilcoxon signed-rank post hoc test with Bonferroni correction) and Kruskal-Wallis test (>2 unrelated samples; Wilcoxon rank-sum test post hoc test with Bonferroni correction). Differences in proportions were tested using χ^2^ test. For classification of single unit responses to light stimulation, significant firing rate changes were assessed statistically using Wilcoxon signed-rank test. Data are represented as median and inter-quartile range. Outliers were removed when their distance from 25^th^ or 27^th^ percentile exceeded 1.5 times the inter-quartile-range.

**References**

1. Suter BA, O'Connor T, Iyer V, Petreanu LT, Hooks BM, Kiritani T, et al. Ephus: Multipurpose Data Acquisition Software for Neuroscience Experiments. Frontiers in Neural Circuits. 2010;4:100.

2. Stujenske JM, Spellman T, Gordon JA. Modeling the Spatiotemporal Dynamics of Light and Heat Propagation for In Vivo Optogenetics. Cell reports. 2015;12(3):525-34.

3. Tehovnik EJ, Sommer MA. Effective spread and timecourse of neural inactivation caused by lidocaine injection in monkey cerebral cortex. Journal of neuroscience methods. 1997;74(1):17-26.

4. Cichon NB, Denker M, Grün S, Hanganu-Opatz IL. Unsupervised classification of neocortical activity patterns in neonatal and pre-juvenile rodents. Frontiers in Neural Circuits. 2014;8.

5. Nolte G, Bai O, Wheaton L, Mari Z, Vorbach S, Hallett M. Identifying true brain interaction from EEG data using the imaginary part of coherency. Clinical Neurophysiology. 2004;115(10):2292-307.

6. Davies DL, Bouldin DW. A cluster separation measure. IEEE transactions on pattern analysis and machine intelligence. 1979;1(2):224-7.

7. Späth H. Cluster Analysis Algorithms for Data Reduction and Classification of Objects. Chichester: Ellis Horwood; 1980.

8. Brockmann MD, Poschel B, Cichon N, Hanganu-Opatz IL. Coupled oscillations mediate directed interactions between prefrontal cortex and hippocampus of the neonatal rat. Neuron. 2011;71(2):332-47.

9. Siapas AG, Lubenov EV, Wilson MA. Prefrontal phase locking to hippocampal theta oscillations. Neuron. 2005;46(1):141-51.

10. Vinck M, van Wingerden M, Womelsdorf T, Fries P, Pennartz CM. The pairwise phase consistency: a bias-free measure of rhythmic neuronal synchronization. NeuroImage. 2010;51(1):112-22.
